# A Role for Early-Phase Transmission in the Enzootic Maintenance of Plague

**DOI:** 10.1371/journal.ppat.1010996

**Published:** 2022-12-15

**Authors:** Cedar L. Mitchell, Ashley R. Schwarzer, Adélaïde Miarinjara, Clayton O. Jarrett, Angela D. Luis, B. Joseph Hinnebusch

**Affiliations:** 1 Laboratory of Bacteriology, Rocky Mountain Laboratories, National Institute of Allergy and Infectious Diseases, National Institutes of Health, Hamilton, Montana, United States of America; 2 Department of Ecosystem and Conservation Sciences, University of Montana, Missoula, Montana, United States of America; University of Pennsylvania, UNITED STATES

## Abstract

*Yersinia pestis*, the bacterial agent of plague, is enzootic in many parts of the world within wild rodent populations and is transmitted by different flea vectors. The ecology of plague is complex, with rodent hosts exhibiting varying susceptibilities to overt disease and their fleas exhibiting varying levels of vector competence. A long-standing question in plague ecology concerns the conditions that lead to occasional epizootics among susceptible rodents. Many factors are involved, but a major one is the transmission efficiency of the flea vector. In this study, using *Oropsylla montana* (a ground squirrel flea that is a major plague vector in the western United States), we comparatively quantified the efficiency of the two basic modes of flea-borne transmission. Transmission efficiency by the early-phase mechanism was strongly affected by the host blood source. Subsequent biofilm-dependent transmission by blocked fleas was less influenced by host blood and was more efficient. Mathematical modeling predicted that early-phase transmission could drive an epizootic only among highly susceptible rodents with certain blood characteristics, but that transmission by blocked *O*. *montana* could do so in more resistant hosts irrespective of their blood characteristics. The models further suggested that for most wild rodents, exposure to sublethal doses of *Y*. *pestis* transmitted during the early phase may restrain rapid epizootic spread by increasing the number of immune, resistant individuals in the population.

## Author summary

The ecology of bubonic plague is complex but depends largely on flea-borne transmission. Certain susceptible rodents experience periodic epizootics that can decimate local populations, but the conditions that lead to these episodes are not fully understood. Fleas can transmit *Yersinia pestis*, the bacterial agent of plague, during two different phases: an early phase within the first few days after their infectious blood meal and again sometime later after the development of a *Y*. *pestis* biofilm in the flea foregut. The relative contribution of these two transmission modes to plague ecology has not been systematically examined. Our results indicate that in most ecological contexts early-phase transmission is too inefficient to drive an epizootic, but instead acts to reduce the number of susceptible individuals in a population, thereby favoring a more stable enzootic state.

## Introduction

Plague is a vector-borne zoonotic disease that primarily afflicts rodents, notably members of the Muridae (rats, mice, gerbils) and Sciuridae (squirrels, marmots, prairie dogs), and persists in extensive natural foci throughout the world. *Yersinia pestis*, the plague bacillus, circulates within these host populations via rodent-flea-rodent transmission cycles that involve several flea vector species. Plague exhibits an enigmatic pattern in which dramatic epizootics in highly susceptible rodent populations periodically flare up from the normal relatively quiescent enzootic background state of a plague focus or from reintroduction of *Y*. *pestis* into a population. The ecological factors and biological mechanisms that initiate and drive these periodic epizootics versus those that tend toward enzootic maintenance have been subjects of long-standing interest [1–5]. Metapopulation structure, fluctuations in population densities of rodents and fleas, and the immune status and degree of susceptibility to overt disease within and among reservoir host species have been theorized to be important [1, 6–12].

One crucial component of plague cycle dynamics is the transmission rate from infected to uninfected hosts. Although it can sometimes be transmitted by direct contact, ingestion, or aerosol, *Y*. *pestis* has evolutionarily adapted to the flea-borne transmission route on which it now depends. Fleas can transmit *Y*. *pestis* in three stages following an infectious blood meal. Transmission can occur the very next time they feed during the first week after infection, a phenomenon referred to as early-phase transmission. The second and third stages of transmission are effectuated after *Y*. *pestis* forms a bacterial biofilm in the proventriculus, a valve between the esophagus and midgut. As the biofilm grows it gradually restricts the passage of blood through the proventriculus and impedes valvular function. In this partially blocked state, fleas are able to ingest some blood but because the proventricular valve is unable to close completely, blood contaminated with bacteria from the flea digestive tract can backflow into the bite site [13, 14]. Eventually, in the final stage, the biofilm can fill the entire proventriculus and completely block the flow of incoming blood into the midgut. When such completely blocked fleas attempt to feed, blood flowing into the esophagus is stopped in front of the blocked proventriculus, the esophagus initially expands, and then the blood, mixed with some of the bacteria washed from the proventricular biofilm, is recoiled back into the bite site [14, 15]. Early-phase transmission was historically referred to as mass transmission, because it is rarely observed unless groups of 5 to 10 or more infected fleas feed simultaneously on a naïve animal. Although long assumed to be a form of mechanical transmission, early-phase transmission also appears to occur via regurgitation of bacteria from a heavily infected proventriculus even though it does not depend on formation of a mature biofilm [14, 16–18]. Thus, bacterial obstruction of the lumen of the proventricular valve and its normal function, to a greater or lesser extent, is the underlying mechanism common to all three modes of transmission [14].

The several rodent flea species implicated in plague transmission cycles vary in their vector efficiency [19]. However, the relative efficiency of the different phases of transmission by individual fleas has not been empirically evaluated in a systematic way. Early-phase transmission and transmission by blocked fleas have been studied in separate experiments and comparisons made between them, but significant differences in experimental design among the studies make the conclusions problematic. Different infectious doses and blood meal sources, both of which greatly affect flea infection and transmission outcomes, have been employed. Transmission by partially blocked fleas has for the most part been neglected; often the blockage rate has been used as the surrogate indicator for transmission beyond the early phase. Mathematical modeling based on parameterization of these available data has supported a role for early-phase transmission in driving epizootics in some ecological contexts [20, 21]. However, there is a recognized need to reexamine the relative vector competency of different flea species and efficiencies of the different transmission modes using newer standardized, more exacting methods [22].

In this study, we examined transmission of *Y*. *pestis* by the North American ground squirrel flea, *Oropsylla montana*. Several aspects of transmission dynamics were recorded during a one-month period following a single infectious mouse or rat blood meal, including infection and mortality rates and the incidence of proventricular blockage. The number of *Y*. *pestis* transmitted by individual fleas during the early phase (2 to 4 days after infection) and after the development of partial or complete blockage was also determined. The data were used to parameterize a simple deterministic susceptible-exposed-infected-recovered (SEIR) model of plague transmission dynamics that we developed. Simulations using the model provided an assessment of the relative contributions of early-phase transmission and biofilm-dependent transmission to disease incidence ensuing from a single cohort of fleas that feed on a highly bacteremic host. The influence of infectious blood source (mouse vs. rat) on plague transmission dynamics was also evaluated.

## Results

### Quantitative evaluation of flea infection and transmission parameters

**Fig 1:**
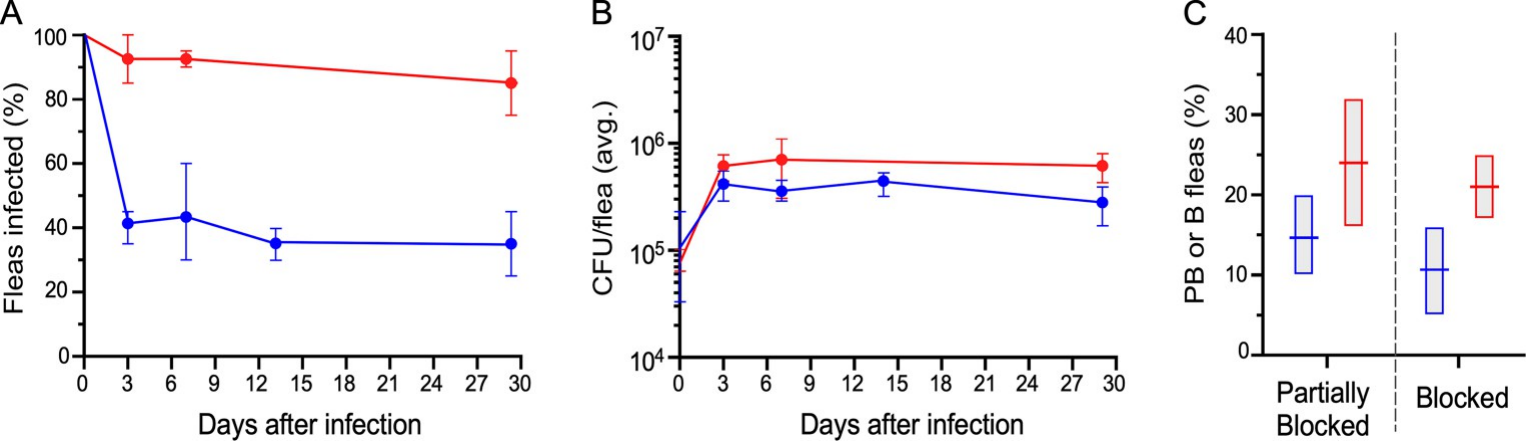
Infection and blockage rates of *O*. *montana* fleas during a four-week period following a blood meal containing 5 to 8 x 10^8^ *Y*. *pestis*/ml in mouse blood (blue symbols) or rat blood (red symbols). (**A**) The percentage of fleas still infected and (**B**) the bacterial load per infected flea at different times after infection. **(C)** The percentage of fleas that developed partial or complete proventricular blockage during the four-week period. The mean and range of three independent experiments using mouse blood and two experiments using rat blood (Table 1) are indicated.

**Fig 2:**
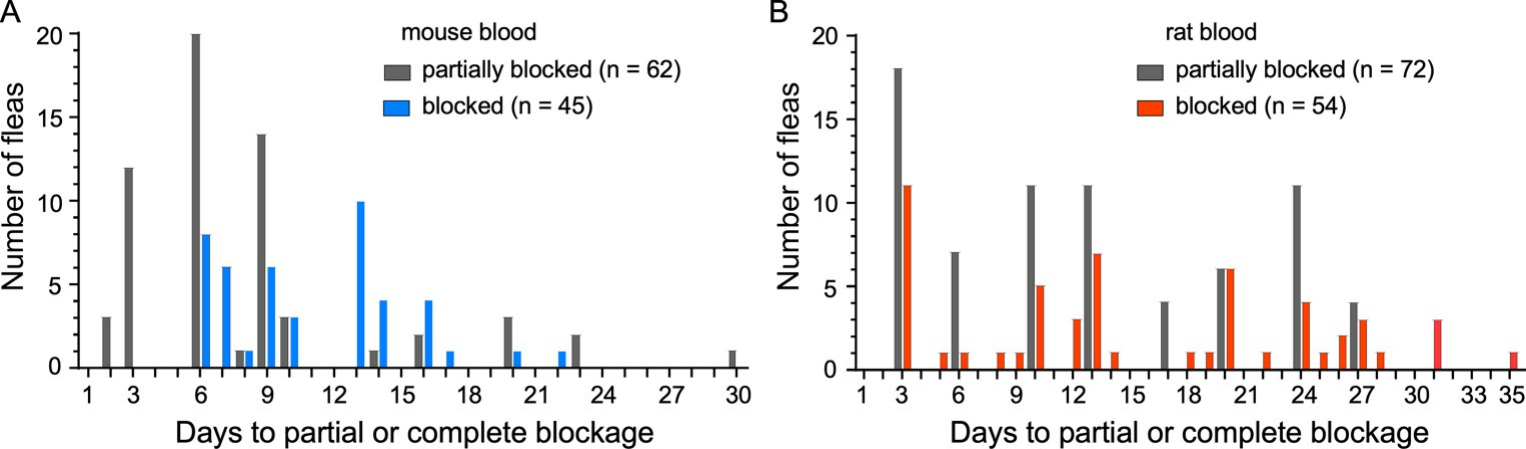
Temporal distribution of the occurrence of partial and complete blockage in *O*. *montana* fleas following a single infectious blood meal containing 5 to 8 x 10^8^ *Y*. *pestis*/ml in mouse or rat blood. The cumulative numbers from the three experiments using mouse blood (**A**) and two experiments using rat blood (**B**) are shown; see Table 1 for details.

Cohorts of *O*. *montana* fleas were infected by feeding on highly bacteremic mouse or rat blood (containing 5 to 8 x 10^8^ *Y*. *pestis*/ml) and thus began with a 100% infection rate at an average of 3 to 6 x 10^4^ *Y*. *pestis* per flea on day 0. Fleas were maintained at 21°C and provided twice-weekly mouse or rat sterile blood meals for 4 weeks and monitored for mortality, infection, and proventricular blockage status (Fig 1). Host blood source had a large effect on infection rate. Consistent with a previous study [23], over half of the fleas infected using mouse blood had completely cleared the infection by day 3. In contrast, around 90% of fleas infected using rat blood remained infected (Fig 1A). The bacterial load of the chronically infected fleas was similar, however (Fig 1B). In correlation with their higher infection rate, a higher percentage of fleas infected using rat blood developed proventricular blockage during the experiments than fleas infected using mouse blood (21% and 11%, respectively; Table 1, Fig 1C). Fleas that appeared to be partially or even completely blocked were seen as early as 2 to 3 days after infection, although the highest incidence occurred between 1 and 3 weeks after infection (Fig 2).

**Table 1:** Values for flea infection and transmission factors determined in this study.

	mouse blood	rat blood
Fleas cleared of infection by:		
day 3	59% (55–65%)	8% (0–15%)
day 7	57% (40–70%)	8% (5–10%)
day 28	65% (55–75%)	15% (5–25%)
Fleas partially blocked (%)	15% (10–21%)	24% (16–32%)
Days to develop partial blockage	8 (2–30)	12 (3–27)
Life span of partially blocked fleas (days) ^a^	7 (1–18)	8 (1–20)
Fleas blocked (%)	11% (5–16%)	21% (17–25%)
Days to develop blockage	11 (6–22)	15 (3–35)
Life span of blocked fleas (days)^a^	5 (1–11)	4 (1–8)
Mortality of unblocked fleas (28 days P.I.)	17% (12–21%)	35% (28–39%)
Transmission efficiency^b^ of:		
Early-phase fleas (day 2–4)	3% (1/38)	10% (4/42)
Partially blocked fleas	11% (2/18)	10% (5/48)
Blocked fleas	50% (20/40)	67% (46/69)
Median no. CFU transmitted^c^ by:		
Early-phase fleas (day 2–4)	1	17
Partially blocked fleas	1,090	417
Blocked fleas	230	63

The average and range of the results from three independent experiments with *O*. *montana*

fleas infected and maintained using mouse blood (n = 177, 209, 283; 669 fleas total) and two experiments using rat blood (n = 247, 192; 439 fleas total) are shown. Of these, 80–100 were used solely for flea infection rate and bacterial load determinations in each experiment (n = 20 fleas for each of the timepoints); the other 429 (mouse blood experiments) and 279 fleas (rat blood experiments) were used to monitor blockage, mortality, and transmission. The early-phase transmission efficiency (rat blood) was based on two separate experiments (n = 60 and 72 fleas; 132 total) with fleas infected with the *Y*. *pestis* Δ*hmsH* mutant.

^a^ Days from diagnosis of partial or complete blockage to death

^b^ The number of positive transmission events divided by the total number of bites by individual fleas in each category

^c^Values for positive transmission events only

**Fig 3:**
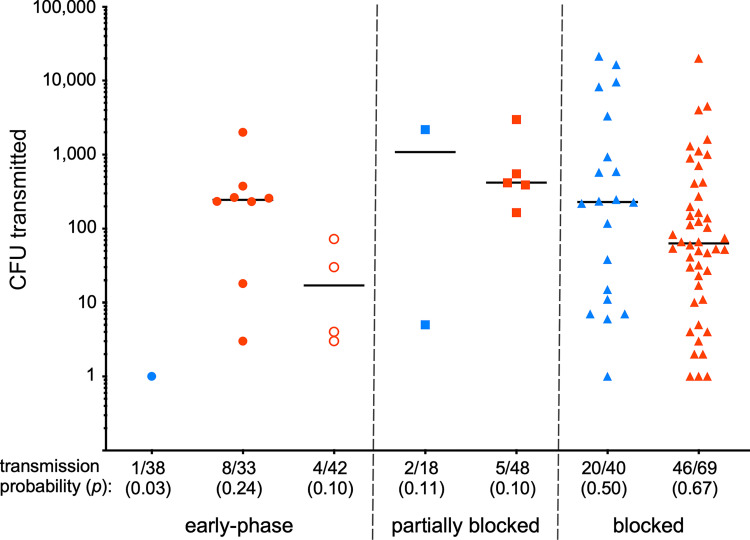
The number of *Y*. *pestis* CFU transmitted by individual *O*. *montana* fleas 2 to 4 days after infection (early-phase) and after the development of partial or complete proventricular blockage. Cumulative results from three experiments using mouse blood (blue symbols), two experiments using rat blood (red symbols), and two experiments using rat blood and the *Y*. *pestis* Δ*hmsH* mutant strain (open circles) are shown (see Table 1 for details); bars indicate the median number of *Y*. *pestis* transmitted per individual flea bite. The transmission probability (number of positive transmissions divided by the total number of trials) is indicated. All early-phase fleas were confirmed to have been infected when they fed for the transmission test. For both the partially blocked and blocked groups, differences in transmission probability and the number of CFU transmitted by fleas infected using mouse blood or rat blood were not statistically significant.

Transmission efficiency of fleas during the early phase (2 to 4 days after the infectious blood meal) and of partially and completely blocked fleas was determined by allowing individual fleas to feed on a small reservoir of sterile mouse or rat blood for 1 h, and then recovering and plating the blood for CFU (colony-forming unit) count. Fleas were examined for evidence of feeding and for proventricular blockage status immediately after the 1 h access period. The rodent source of the infectious blood meal had a large effect on early-phase transmission efficiency. Only 1 of 38 fleas (3%) infected using mouse blood transmitted during their first blood meal 2 to 4 days later, and only 1 CFU was recovered from the blood that this flea fed upon (Fig 3). In contrast, 8 of 33 (24%) of fleas infected using rat blood transmitted 3 to 2,000 CFU during early-phase transmission trials. Notably, however, 5 of these 8 fleas were observed to be completely blocked and 2 appeared to be partially blocked after this initial post-infection feeding on day 3 or 4. For this reason, we repeated early-phase transmission trials with fleas infected with a *Y*. *pestis hms* mutant strain, which is unable to block fleas but is fully transmissible in the early phase [17, 24]. To clearly distinguish the two transmission modes, we concentrated on results with this *hms* mutant [4 of 42 fleas (10%) transmitted 3 to 72 CFU (median 17 CFU); Fig 3] to estimate the early-phase transmission efficiency.

Our estimate of early-phase transmission efficiency for fleas infected using rat blood was in line with those reported previously for *O*. *montana* infected similarly that were used to challenge mice [17, 20, 25–27]. However, our estimate for fleas infected using mouse blood (3%) was much lower than we found with the mouse challenge model (18%; [27]). This may in part be due to a lower sensitivity of the *in vitro* transmission model. The number of CFUs transmitted in the early phase by fleas infected using mouse blood was clearly very low, but some transmissions of one or a few bacteria may have been missed because the efficiency of plating and recovering very low CFU numbers from a blood reservoir is likely not 100%. For the early-phase probability of transmission parameters (*p*_ep_) described below, therefore, we chose to use the higher estimates reported for the mouse challenge model (18% and 14% for *O*. *montana* infected using mouse or rat blood, respectively) [27]. These parameter values were used in conjunction with our empirical data on the number of wild-type and *hms* mutant *Y*. *pestis* transmitted by individual early-phase fleas infected using mouse or rat blood, respectively (Fig 3).

**Fig 4:**
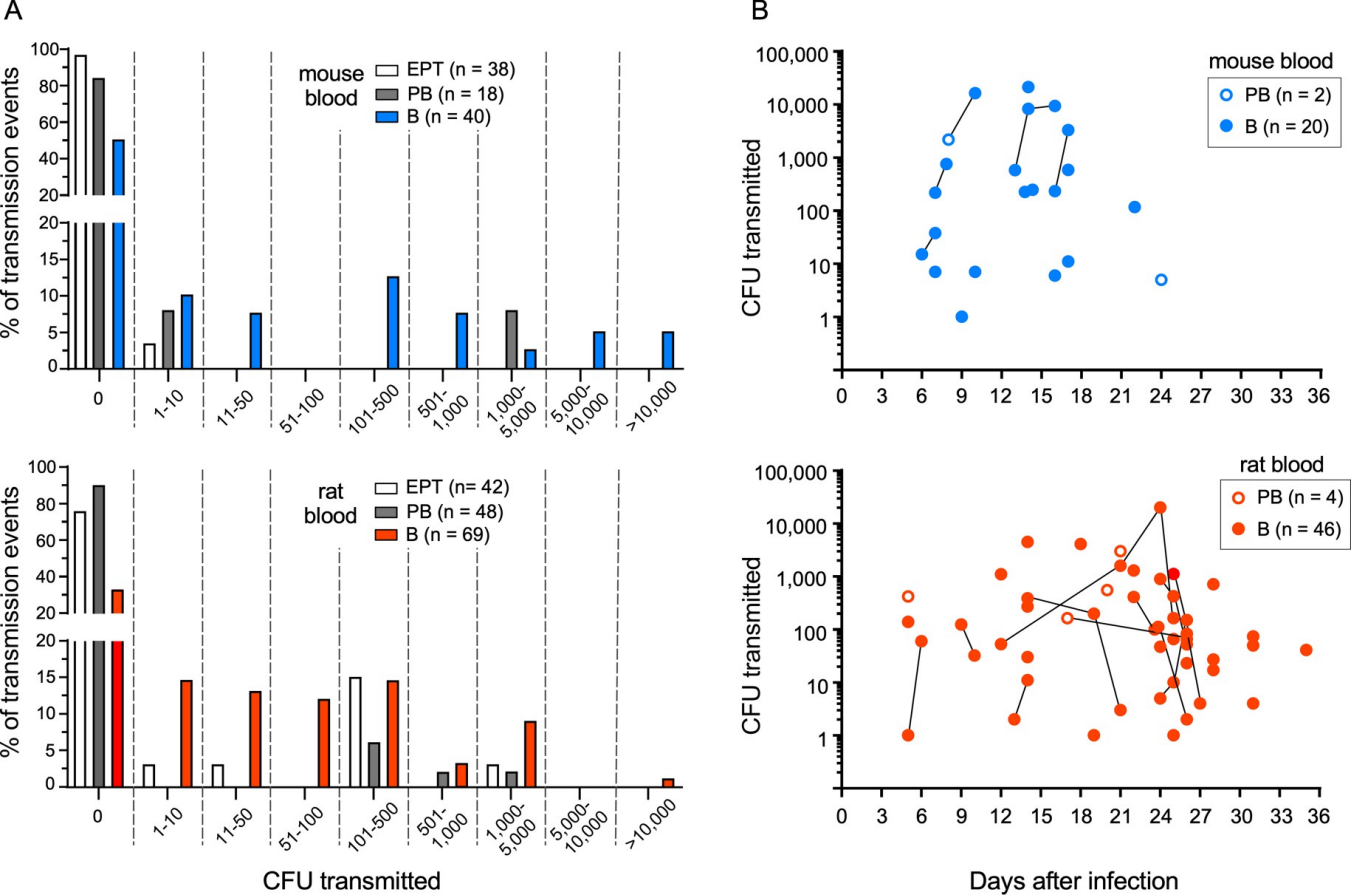
(**A**) Frequency distribution histogram of the numbers of *Y*. *pestis* CFU transmitted by individual *O*. *montana* fleas during the early phase (EPT) and by partially blocked (PB) and completely blocked (B) fleas. (**B**) Temporal distribution pattern of the number of CFU transmitted by individual partially blocked (PB) and completely blocked (B) fleas (positive transmission events only). Lines connecting data points indicate transmissions by the same flea on successive days. Cumulative results from three experiments using mouse blood (blue symbols) and two experiments using rat blood (red symbols) are shown; see Table 1 for details.

Transmission by partially blocked fleas was also rather inefficient–only about 10% of feeding events resulted in transmission, regardless of the infectious blood source (Fig 3). Completely blocked fleas had the highest transmission efficiency, and it was similar for fleas infected using mouse or rat blood (50% and 67%, respectively; *p* = 0.1). Transmission tests were repeated periodically for blocked fleas as long as they remained alive, and several of these fleas transmitted on more than one day (Fig 4). In one case, a single blocked flea transmitted four different times over a period of 12 days.

Another aspect of transmission efficiency is the number of bacteria transmitted per bite, which we were able to assess on an individual flea basis. The number of *Y*. *pestis* transmitted by blocked fleas can be highly variable [28, 29]. Our results confirm that, with a range from 1 to >10,000 CFU transmitted by individual fleas, and also indicate that the number of *Y*. *pestis* transmitted by early-phase and partially blocked fleas can vary widely (Figs 3 and 4) An exception may be early-phase transmission by fleas infected using mouse blood, which was of such low efficiency that only small numbers are likely to be transmitted (Fig 3). A summary of the results of the flea infection and transmission experiments is given in Table 1.

### Model simulations of flea-borne transmission dynamics

**Fig 5:**
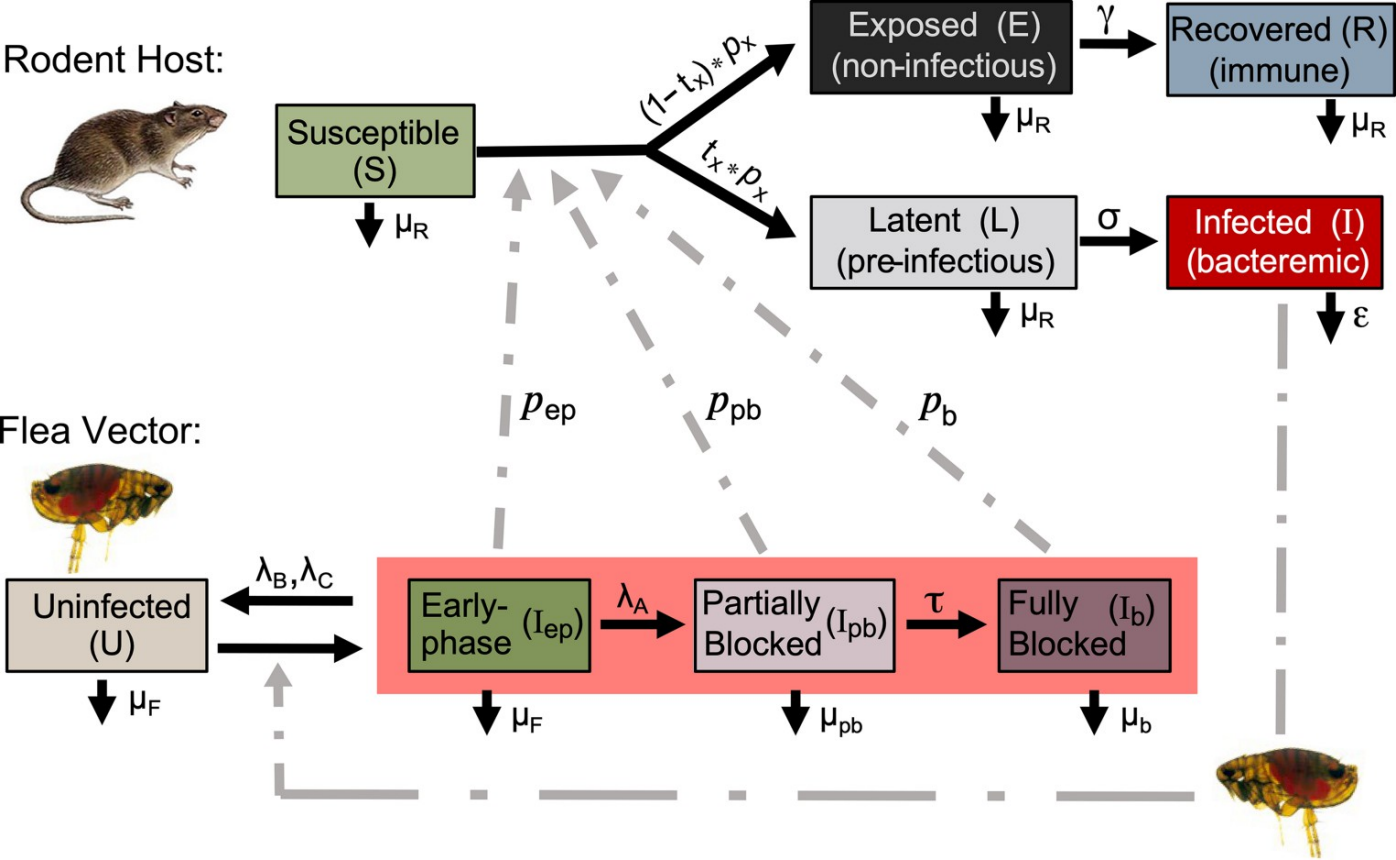
Flow chart of the flea vector-rodent host model. The three transmission-competent stages of flea infection are highlighted, and the different possible outcomes following transmission to the rodent host are indicated. See text and Tables 2 and 3 for details. *t*_*x*_ = *t*_ep_, *t*_pb_, or *t*_b_.; *p*_*x*_ = *p*_ep_, *p*_pb_, or *p*_b_.

**Fig 6:**
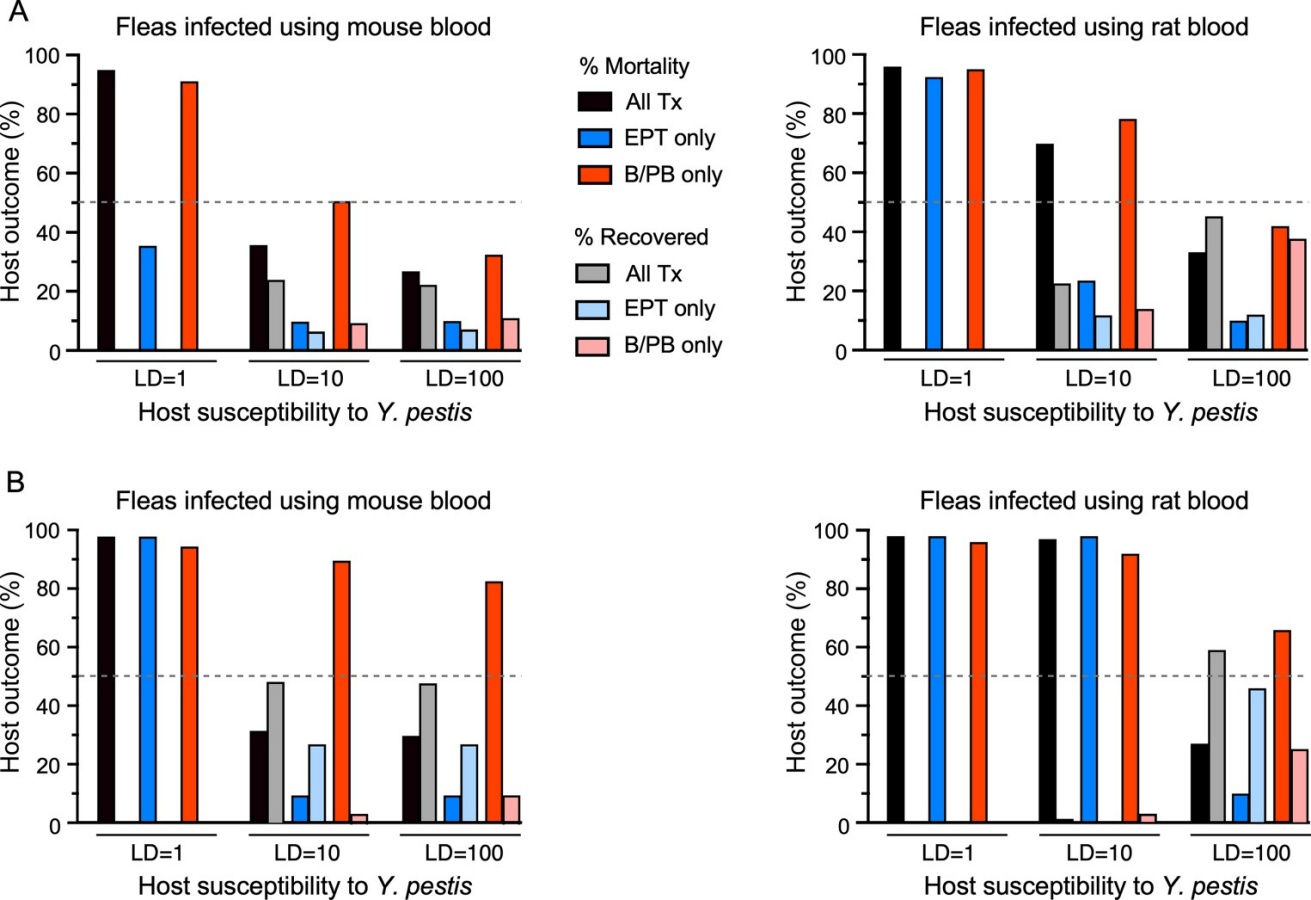
Model predictions of the incidence of infected-dead (% mortality) and infected-recovered (% recovered) hosts in populations with different levels of susceptibility [lethal dose (LD) of 1, 10, or 100 *Y*. *pestis* CFU]. Separate outcomes produced by fleas infected using mouse blood or rat blood in which both early-phase transmission and biofilm-dependent transmission by partially and completely blocked fleas are operative (All Tx); or in which only early-phase transmission (EPT only) or only biofilm-dependent transmission (B/PB only) are operative are indicated. All simulations were initiated with 9 susceptible hosts, 1 infected (highly bacteremic) host, and 50 uninfected fleas, monitored over a 100-day period. The results from two versions of the model using (**A**) unmodified parameters and (**B**) modified parameters for probability of transmission (*p*) and probability of transmission at or above a lethal dose (*t*) that account for cumulative transmission by simultaneous flea bites (S1 and S2 Figs). See text for details.

We developed a deterministic SEIR model to compare the relative contribution of three transmission stages on plague dynamics in the context of two host blood sources that are known to influence infection of fleas and early-phase transmission [16, 30]. Conceptual design of the model is illustrated in Fig 5. Our experimental results summarized above were used to estimate the parameter values of the flea vector submodel (Table 2). Parameter values for the rodent host submodel (Table 3) are from published sources. We examined different model structures based on fleas infected using bacteremic mouse or rat blood, host susceptibility to fatal plague [lethal dose (LD_100_) of 1, 10, or 100 *Y*. *pestis* CFUs], and transmission mode (early-phase transmission or biofilm-dependent transmission by blocked or partially blocked fleas). The starting condition was 9 susceptible hosts, 1 infected (highly bacteremic) host, and 50 uninfected fleas; model outputs were recorded over a simulated 100-day period (S1 Fig). Fig 6A shows the predicted mortality for the various scenarios. With all transmission modes operative, plague epizootics (arbitrarily defined as ≥ 50% cumulative mortality during the 100-day simulation) ensued in the most susceptible host population, but at the second tier of susceptibility (LD = 10 CFU) the output was host-blood dependent. Fleas infected using rat blood were predicted to cause an epizootic, but fleas infected using mouse blood were not. In the more resistant population (lethal dose of 100 CFU), enzootic scenarios (<50% mortality) ensued with both sets of fleas. Most of the mortality was attributable to transmission by blocked fleas–when early-phase transmission parameters were set to zero, mortality was equivalent or even slightly higher than when both transmission modes were operative. Conversely, when blockage-dependent transmission was removed, predicted mortality was 2.7- to 3.6-fold lower in all cases. The strong influence of the infectious blood source on early-phase transmission (Fig 3) was reflected in the model output (Figs 6A and S1). Early-phase-dependent mortality effected by fleas infected using mouse blood was 36% in the highly susceptible host population (LD = 1 CFU) but nil in the other two populations–the only fatality was the single infected host used to initiate the model simulations. In contrast, early-phase transmission generated higher mortality (93% and 24%) in the two susceptible populations in the rat blood context, although it was still negligible in the more resistant population (LD = 100 CFU).

**Table 2:** Summary of parameter values, flea vector sub-model.

Parameter	Definition	Calculation	Value		Ref.
			Mouse blood	Rat blood	
**b**	Daily biting rate (uninfected, early-phase, and partially blocked fleas)	$\frac{1}{\begin{array}{c} \;avg.\;interval\;(days)\\ between\;feeds \end{array}}$	0.4	0.4	This study
**b**_**b**_	Daily biting rate (blocked flea)	$\frac{1}{\begin{array}{c} \;avg.\;interval\;(days)\;between\\ feeding\;attempts\;(B\;fleas) \end{array}}$	> 1	> 1	This study, [15, 28]
**α**	Proportion of fleas infected from host with high bacteremia	$\frac{\mathrm{no}.\;\mathrm{fleas}\;\mathrm{infected}\;(d0)}{\begin{array}{c} no.\;fleas\;that\;fed\;on\\ bacteremic\;blood \end{array}}$	1.0	1.0	This study
**λ**_**A**_	Rate of developing partial blockage	$\frac{(\%\;\mathrm{PB}\;\mathrm{fleas})÷100}{(\mathrm{avg}.\;\mathrm{time}\;\mathrm{to}\;\mathrm{PB})-3d}$	0.035	0.04	This study
**λ**_**B**_	Rate of infection clearance	$\frac{(\%\;\mathrm{fleas}\;\mathrm{cleared}\;\mathrm{by}\;3d)÷100}{3d}$	0.20	0.02	This study
**λ**_**C**_	Rate fleas leave infectious population after early phase	$\frac{\begin{array}{c} (\%\;fleas\;still\;infected\;after\;3d\\ but\;never\;PB/B)÷\mathrm{100} \end{array}}{(\mathrm{avg}.\;\mathrm{time}\;\mathrm{to}\;\mathrm{PB})-3d}$	0.07	0.06	This study
**τ**	Rate of developing complete blockage	$\frac{1}{\mathrm{avg}.\;\mathrm{days}\;\mathrm{from}\;\mathrm{PB}\;\mathrm{to}\;B}$	0.39	0.48	This study
**μ**_**f**_	Uninfected flea mortality rate	$\frac{1}{\mathrm{avg}.\;\mathrm{life}\;\mathrm{span}\;\mathrm{of}\;\mathrm{fleas}\;(\mathrm{days})}$	0.02	0.02	[31]
**μ**_**pb**_	Mortality rate of partially blocked fleas	$\frac{1}{\mathrm{avg}.\;\mathrm{TTD}\;\mathrm{after}\;\mathrm{becoming}\;\mathrm{PB}}$	0.14	0.13	This study
**μ**_**b**_	Mortality rate of blocked fleas	$\frac{1}{\mathrm{avg}.\;\mathrm{TTD}\;\mathrm{after}\;\mathrm{becoming}\;B}$	0.20	0.26	This study
**p**_**ep**_	Probability of early-phase transmission	$\frac{\mathrm{no}.\;\mathrm{transmission}\;\mathrm{events}}{\begin{array}{c} no.\;pos.\;feeding\;attempts\\ \left(d\;2‐4\right) \end{array}}$	0.18 (0.86)	0.14 (0.78)	This study, [27]
**p**_**pb**_	Probability of transmission (partially blocked fleas)	$\frac{\mathrm{no}.\;\mathrm{transmission}\;\mathrm{events}}{\begin{array}{c} no.\;pos.\;feeding\;attempts\\ \left(by\;PB\;fleas\right) \end{array}}$	0.11	0.10	This study
**p**_**b**_	Probability of transmission (blocked fleas)	$\frac{\mathrm{no}.\;\mathrm{transmission}\;\mathrm{events}}{\begin{array}{c} no.\;pos.\;feeding\;attempts\\ \left(by\;B\;fleas\right) \end{array}}$	0.5 (0.75)	0.67 (0.89)	This study
**t**_**ep**_	Proportion of early-phase transmission events ≥ LD of rodent	$\frac{\begin{array}{c} no.\;events\;in\;which\\ CFUs\;transmitted\geq LD \end{array}}{\begin{array}{c} total\;transmission\;events\\ \left(early\;phase\right) \end{array}}$	1.0 (1.0) ^a^	1.0 (1.0) ^a^	This study
			0 (0)^b^	0.5 (1.0)^b^	
			0 (0)^c^	0 (0)^c^	
**t**_**pb**_	Proportion of transmission events by partially blocked fleas ≥ LD of rodent	$\frac{\begin{array}{c} no.\;events\;in\;which\\ CFUs\;transmitted\geq LD \end{array}}{\begin{array}{c} total\;transmission\;events\\ \left(by\;PB\;fleas\right) \end{array}}$	1.0 ^a^	1.0^a^	This study
			0.5^b^	1.0^b^	
			0.0^c^	1.0^c^	
**t**_**b**_	Proportion of transmission events by blocked fleas ≥ LD of rodent	$\frac{\begin{array}{c} no.\;events\;in\;which\\ CFUs\;transmitted\geq LD \end{array}}{\begin{array}{c} total\;transmission\;events\\ \left(\mathrm{by}\;B\;\mathrm{fleas}\right) \end{array}}$	1.0 (1.0) ^a^	1.0^a^	This study
			0.8 (0.96)^b^	0.8 (0.96)^b^	
			0.65 (0.88)^c^	0.41 (.65)^c^	

B, blocked; PB, partially blocked; TTD; time to death.

Values in parentheses adjusted to account for cumulative transmission by simultaneous flea bites as described in Methods section.

^a, b, c^ = Values for LD (lethal dose_100_) = 1, 10, or 100 *Y*. *pestis* CFU

**Table 3:** Summary of parameter values, rodent host sub-model.

Parameter	Definition	Calculation	Value*	Ref.
**γ**	Recovery rate after infectious transmission	$\frac{1}{avg.\;time\;to\;recovery\;(14\;d)}$	0.07	[32]
**σ**	Rate of developing terminal bacteremia after infectious transmission	$\frac{1}{avg.\;time\;to\;terminal\;bacteremia\;(4\;d)}$	0.25	[33–38]
**μ**_**R**_	Mortality rate of uninfected rodents and those with latent or resolved infections	$\frac{1}{\mathrm{avg}.\;\mathrm{life}\;\mathrm{span}\;\mathrm{of}\;\mathrm{rodent}}$	0.002	[39]
**ε**	Mortality rate of rodents with septicemic plague	$\frac{1}{\begin{array}{c} avg.\;life\;span\;of\;rodent\;\\ with\;terminal\;bacteremia\;(2\;d) \end{array}}$	0.50	[28, 33]

Parameter values are based on data from mouse and rat models of acute plague

Recent studies of early-phase transmission efficiency have used a model in which groups of ~10 fleas infected using highly bacteremic rat blood fed simultaneously on highly susceptible laboratory mice (LD_50_ <10 CFU) [20, 40, 41]. To explicitly examine this scenario, we adjusted our early-phase transmission parameters, which are based on an individual flea bite, to reflect the cumulative probability of 10 simultaneous flea bites transmitting a lethal dose. In addition, since a blocked flea makes repeated feeding attempts, we likewise adjusted the blocked flea transmission parameters to reflect the cumulative probability of transmitting a lethal dose in two consecutive feeding attempts. The adjusted parameters are listed in Table 2. In this scenario, early-phase transmission by fleas infected using mouse blood was sufficient to produce an epizootic only in the most susceptible host population, and in populations with the first two levels of susceptibility by fleas infected using rat blood (Figs 6B and S2). High mortality ensued in the more resistant host population only when transmission by blocked and partially blocked fleas was included in the model. Notably, with both modes of transmission active, mortality in this more resistant population was less than half that (27%) of the mortality predicted when blockage-dependent-transmission operated alone (early-phase transmission parameters set to zero; 66% mortality). Correspondingly, infected but recovered hosts were greater in this population when both modes of transmission were operative than when early-phase transmission was eliminated (59% and 25% recovery rate, respectively; Figs 6 and S2).

### Estimation of flea densities required for enzootic and epizootic transmission dynamics

**Fig 7:**
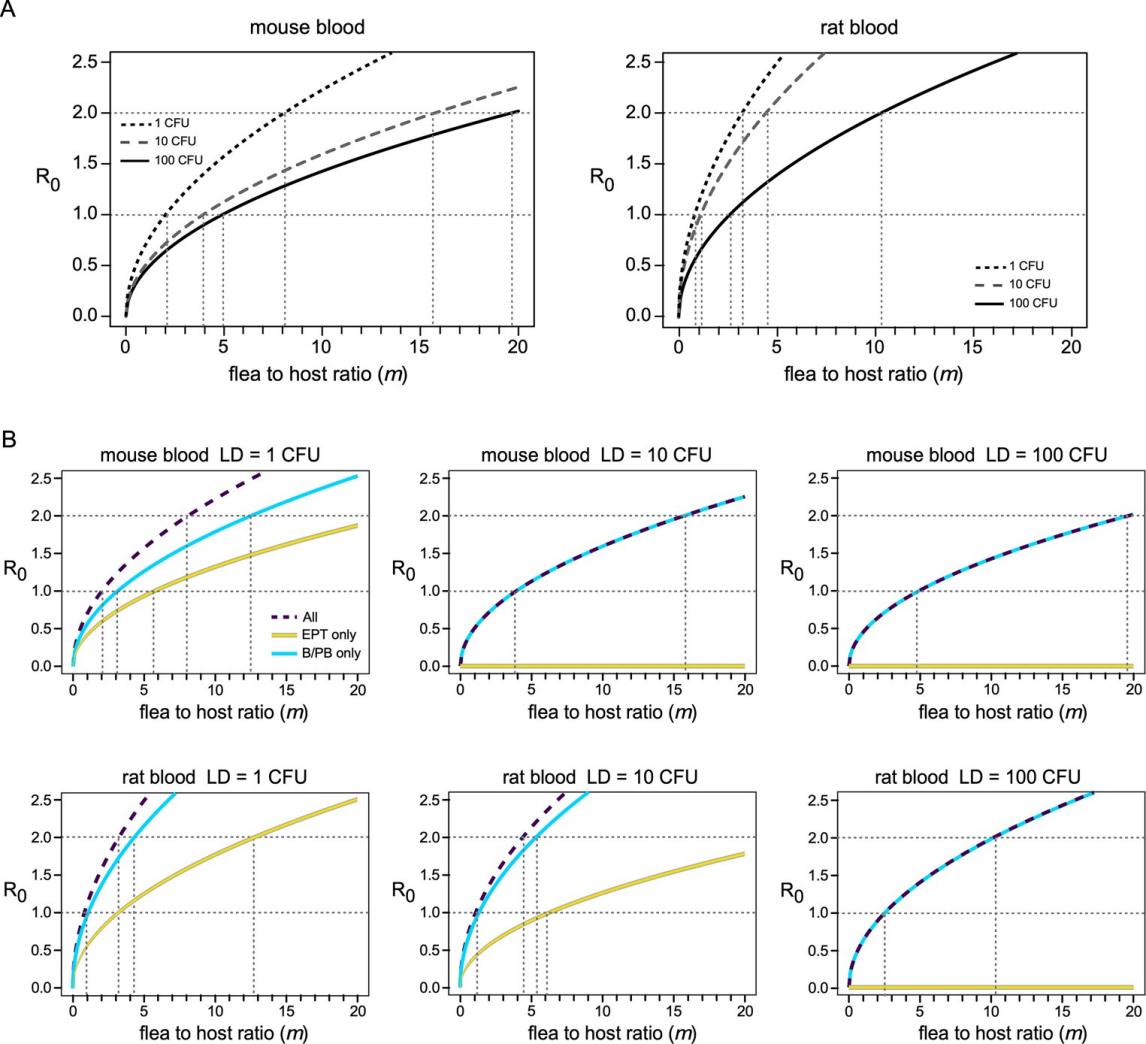
The number of *O*. *montana* fleas per host (*m*) required to realize different levels of host-to-host transmission (R_0_) predicted by the SEIR model, with all transmission modes operative (**A**); or (**B**), with only early-phase transmission (EPT only) or only biofilm-dependent transmission (B/PB only) operative, shown relative to each other and to cumulative transmission by both modes (All). The results for fleas infected using mouse blood or rat blood and for three host populations with different susceptibilities to *Y*. *pestis* (lethal dose of 1, 10 or 100 CFU) are shown. Dashed lines indicate the intersects of the curves for R_0_ = 1 (enzootic) and R_0_ = 2 (epizootic) conditions.

The basic reproduction number (R_0_) is defined as the number of secondary cases that ensue from a single case in a naïve population. We used the next-generation matrix method [42] on our SEIR model to estimate R_0_ values for the various scenarios and to estimate the flea density per host (*m*) that would be required to sustain an enzootic or epizootic state (defined here as R_0_ = 1 and R_0_ = 2, respectively). With all transmission modes in effect, an *m* value of five or fewer fleas per host was sufficient to achieve an enzootic state (R_0_ = 1) for all the scenarios (Fig 7A). To reach an epizootic state (R_0_ = 2), *m* values of 8, 16, and 20 were estimated from the model, respectively, for fleas infected using mouse blood with the three host populations (LD of 1, 10, or 100 *Y*. *pestis* CFU). For fleas infected using rat blood, estimated *m* values required for R_0_ = 2 were 4, 5, and 11 (Fig 7A).

Flea densities required for enzootic and epizootic conditions were also estimated separately for early-phase transmission only and for transmission by blocked fleas only. The results from the SEIR model are shown in Fig 7B. In all cases, the flea burdens required for a given R_0_ level were lower for blocked-flea transmission than for early-phase transmission. For example, in a host population with rat blood characteristics for which the LD = 10 *Y*. *pestis* CFUs, 6 fleas per host were sufficient to drive an epizootic (R_0_ = 2) by the proventricular blockage mechanism, whereas early-phase transmission would require ~25 fleas per host. Early-phase transmission alone was not sufficient for even enzootic maintenance–blocked fleas were required (Fig 7B).

## Discussion

In this study we systematically examined flea-borne transmission dynamics during a one-month period following an infectious blood meal. Cohorts of *O*. *montana* fleas were infected uniformly, using two different host blood sources, and then transmission by individual fleas in three progressive stages of transmission competence was evaluated. Two aspects of transmission efficiency were quantified: the probability of transmission and the number of *Y*. *pestis* transmitted during a single flea bite. The standardized experimental design enabled a more stringent head-to-head comparison of the early-phase and proventricular-blockage dependent transmission modes. Previous comparisons have depended on separate studies that were not standardized as to infectious dose, blood source, or the suitability of the experimental conditions for the general fitness of a particular flea species, all of which can affect infection, blockage rates, and transmission dynamics [16, 23, 29, 30, 33, 40, 43]. Early-phase transmission dynamics following rat blood meals containing ~10^9^ *Y*. *pestis*/ml have been compared with blocked-flea transmission dynamics following infection by feeding on guinea pigs with a much lower bacteremia level, such that only a minority of fleas (~20 to 32%) that fed on them became infected [44–46]. This low infection rate is consistent with a bacteremia of only about 10^7^ *Y*. *pestis*/ml [29, 33], below the threshold for efficient early-phase transmission [43]. Another problematic area has been the essentially preliminary, and sometimes discordant, available data on blockage rates of different flea vectors, often based on single trials with small sample sizes performed under a variety of conditions and lacking important controls [22, 40, 44]. For example, we recently showed that the prairie dog flea, *Oropsylla hirsuta*, can become blocked at a rate much higher than previously reported [47]. Other studies incorrectly reported that *O*. *montana* rarely becomes blocked and transmits beyond the early phase [20, 28, 33, 48]. As shown here and in previous studies however, *O*. *montana* readily becomes blocked and transmits efficiently by the proventricular blockage mechanism [23, 49–51]. Blocked *O*. *montana* are efficient vectors and can transmit large doses of *Y*. *pestis* (Fig 3).

The focus of this study was the transmission rate from flea to rodent, which we maximized by infecting the fleas with blood with the high bacteremia level that has been used for early-phase transmission efficiency experiments [20, 52, 53]. Because host blood source can influence infection and early-phase transmission rates [16, 30], we compared fleas infected using mouse blood or rat blood. Fleas in three transmission-competent states were examined individually. Early-phase transmission by fleas infected using mouse blood was rare and inefficient but was much better if the fleas were infected using rat blood. These results are consistent with a previous study that evaluated early-phase transmission by groups of fleas [16]. Rat blood promotes early-phase transmission because it induces a phenomenon termed post-infection esophageal reflux (PIER), in which a mixture of partially digested blood, hemoglobin crystals, and *Y*. *pestis* is refluxed into the foregut soon after an infectious blood meal [16]. Mouse blood, characterized by a more soluble hemoglobin molecule and a faster digestion rate in the flea gut, does not engender PIER. As in the previous study [16], some fleas appeared to be completely blocked as early as three days after infection using rat blood, suggesting that early-phase can overlap temporally with biofilm-dependent transmission (Fig 2). For this reason, we based the early-phase transmission estimate on fleas infected with a *Y*. *pestis hms* mutant, which is incapable of producing proventricular blockage but fully capable of early-phase transmission [17, 24].

A lower percentage of fleas infected using mouse blood developed partial or complete blockage (Fig 1); but this can be attributed to their lower infection rate compared to fleas infected using rat blood, consistent with results reported previously [23]. Transmission by partially blocked fleas was surprisingly inefficient, comparable to early-phase transmission, which may be because the transmission mechanisms are more or less the same (transient or partial impedance of blood feeding), although partial blockage, unlike early-phase, develops later and is biofilm-dependent [14, 16, 18]. However, no effect of blood source on transmission by partially blocked fleas was evident. Transmission by completely blocked fleas was the most efficient of the three stages, both in terms of transmission rate and the number of CFUs transmitted per flea (Fig 3). At this stage also, no difference was apparent between blocked fleas infected using mouse or rat blood. After their 1-h feeding attempts, 50 to 67% of individual blocked fleas had transmitted, and ~10% of these transmitted >1,000 CFUs, with >10,000 CFU recovered in three instances. On average, the number of CFU transmitted by a blocked *O*. *montana* flea is greater than the number transmitted by a blocked *Xenopsylla cheopis* flea [23, 29].

A second aspect of this study was to use the experimentally derived transmission efficiency data to parameterize an SEIR model of plague dynamics that we developed. Here the main goal was to compare the relative importance and contribution of the early-phase and proventricular blockage-dependent transmission modes in determining epizootic outbreaks. The model outputs indicated that blockage-dependent transmission was most important in producing epizootics, regardless of host blood source. Our modeling indicated that early-phase transmission alone resulted in significantly less mortality, and a large effect of host blood source was evident. Early-phase fleas infected using rat blood could drive an epizootic, but only in the most susceptible host population (LD = 1 CFU). Early-phase transmission by fleas infected using mouse blood was insufficient by itself to produce epizootic conditions in any host population.

Model parameter values were estimated on an individual flea bite basis. However, a feature of flea-borne transmission in nature is that several infected fleas feeding simultaneously on a single host in the early-phase window, or a blocked flea making repeated feeding attempts in succession can produce an augmented, cumulative transmission efficiency. For example, a blocked flea will bite continuously and persistently in the few days before it dies from starvation; and blocked flea transmission efficiency estimates based on single, short term exposure trials are acknowledged to be underestimates [8, 28]. The probability of a blocked flea cumulatively transmitting significant numbers of CFUs in repeated bites likely approaches 100%. In contrast, early-phase transmission is primarily limited to the first feeding after infection, with reduced transmission during later feeds unless reinfected [52]. Therefore, in a second version of the model we incorporated a specific scenario for effective efficiency of cumulative early-phase transmission (simultaneous bites by 10 fleas modeled) and also of blocked-flea transmission (conservatively, 2 consecutive bites by a single blocked flea modeled). In this version of the model, early-phase transmission by fleas infected using mouse blood was sufficient to drive an epizootic, but only in the most susceptible population (LD = 1 CFU). Early-phase transmission by fleas infected using rat blood was sufficient to drive an epizootic in hosts in which the lethal dose was 10 CFU or less. In either version of the model, however, early-phase transmission was incapable of driving an epizootic in a more resistant population (LD = 100 CFU), which is more representative of most wild rodents. Blockage-dependent transmission was essential for epizootic scenarios in those populations. Notably, predicted mortality increased when early-phase transmission was set to zero, whereas the number of recovered hosts was higher when both transmission modes were operative. This suggests that early-phase transmission would act to dampen outbreaks in more resistant populations because the sublethal doses usually delivered lead to recovery and immunity rather than disease. In a previous experiment in which laboratory mice were fed on by 3 to 12 infected *O*. *montana* early-phase fleas, the majority of transmissions were detected by seroconversion only [27]. Thus, blocked flea transmission is likely the driving force behind epizootics in moderately resistant populations. Based on some prior reports that *O*. *montana* rarely becomes blocked, this mode of transmission was discounted *a priori* in an earlier model of plague dynamics as vectored by this flea [54]. Our results show that this needed to be reevaluated, because in this and a previous study [23] we show that blockage-dependent transmission by *O*. *montana* is more efficient than early-phase transmission. In accordance, another study reported that *O*. *montana* transmits at higher efficiency 7 to 21 days after infection than in the early phase [25].

The higher vector competence of blocked *O*. *montana* fleas was also reflected in the estimated flea burden required to drive an epizootic, which was lower than for early-phase transmission, particularly in a more resistant host population (Fig 7B). The value we estimated for early-phase transmission by *O*. *montana* infected using rat blood and a susceptible population was similar to a previous estimate for *X*. *cheopis* that used the same infection scenario [40]. That study came to an opposite conclusion than we did for blocked flea transmission, maintaining that the flea burden would have to be higher than for early-phase transmission. However, the parameter values for blocked flea transmission were based on a single separate study in which the fleas were infected by feeding on mice with varying levels of bacteremia, such that their blockage rate was only 7% [33]. When *X*. *cheopis* feed on highly bacteremic blood, as used for the early-phase experiments, however, several studies have shown that the blockage rate is 35 to 40% or higher [18, 23, 24, 28, 29, 55–58].

Early-phase transmission, although the first mode of transmission to be described, received relatively little attention after the blockage-dependent transmission mode was discovered. Some, however, proposed that early-phase transmission could add momentum to an epizootic when the plague incidence was high enough to reduce a population size, leading to increased flea burdens on remaining hosts [28, 59]. According to this scenario, the large number of questing fleas that had last fed on a host that died from plague septicemia could transmit when they next fed a new host in a phenomenon called mass transmission (the original name for early-phase transmission, reflecting the fact that many fleas feeding simultaneously are required for a high probability of transmission). Our results also indicate that a high flea burden is required for productive early-phase transmission. Transmission by blocked and partially blocked fleas was still considered to be essential for stable ecological maintenance of flea-borne plague [28, 60]. This premise is supported by the fact that all *Y*. *pestis* strains maintain the genes required for flea biofilm formation, even though they are not required for virulence in mammals [24, 61, 62]. A previous theoretical modeling study also concluded that gaining the ability to block fleas was evolutionarily adaptive for *Y*. *pestis* because transmissibility is superior to that of unblocked (*e*.*g*. early-phase) fleas [63].

More recently, a series of studies concluded that early-phase transmission might be the principal driving force of epizootics, particularly in rodent-flea cycles in which the primary flea vector reportedly develops proventricular blockage at low incidence (reviewed in [21]). These conclusions were based on transmission results with highly susceptible laboratory mice challenged by groups of ~10 fleas that had been infected 1 to 4 days earlier by feeding on highly bacteremic rat blood. Our simulations also indicate that early-phase transmission could be sufficient to drive an epizootic in those conditions. However, early-phase transmission varies with host blood–it is less efficient following infection with bacteremic mouse blood, (Fig 3; [16]), and may be important in driving epizootics only with hosts whose blood, like that of brown rats, induce PIER in fleas. Furthermore, most wild rodents are more resistant to *Y*. *pestis* than laboratory mice. For example, the LD_50_ of *Y*. *pestis* for the ground squirrel hosts of *O*. *montana* is reportedly at least 250 to >1,000 CFU [64–66], although this value is likely to vary locally as well as within a given population. It is not known whether transmission efficiency in conjunction with ground squirrel blood would be more like mouse or rat blood, and it will be important to examine that in future studies. In either case, our results suggest a different ecological role for early-phase transmission within most rodent populations. By transmitting a sublethal dose, early transmission may serve to increase the number of resistant individuals in a population in which plague is introduced, in a sense vaccinating them against an otherwise lethal challenge by the later, more efficient transmission by blocked fleas.

Long-term focal persistence of plague requires stable host-pathogen dynamics to maintain an enzootic state. A pathogen as virulent as *Y*. *pestis*, which depends on producing a lethal, high-density bacteremia in the host to infect its flea vectors would seemingly be at risk of burning through host populations too quickly for stable ecological maintenance. This epizootic scenario can occur in the most susceptible hosts. However, our models suggest that exposure of most wild rodents to sublethal, immunizing doses of *Y*. *pestis* transmitted during the early phase may ameliorate rapid epizootic spread by reducing the number of susceptible individuals in the population. The coexistence of resistant and susceptible hosts in a population has long been thought to be a factor in the enzootic persistence of plague despite its high virulence [11]. Thus, in many situations early-phase transmission may be more important in maintaining the enzootic state than in driving an epizootic. The effect of early-phase transmission in generating partially immune populations would also increase the time span of enzootic prevalence, during which the movement of individuals could spread the disease to new populations in new areas [67].

### Summary

Transmission efficiency of individual *O*. *montana* fleas was measured over a four-week period after they had fed on mouse or rat blood containing 5 to 8 x 10^8^ *Y*. *pestis*/ml. Both early-phase and proventricular blockage modes of transmission were monitored for cohorts of fleas following a single infectious blood meal. The results indicate that: 1) blockage-dependent transmission is much more efficient than early-phase transmission, both in terms of the probability of transmission and the number of CFUs transmitted. The recurrent biting behavior of blocked fleas is a significant force multiplier of transmission [68], as a single blocked flea can cumulatively transmit thousands of CFUs before it dies of starvation. 2) Host blood source strongly affects early-phase transmission efficiency, but not blockage-dependent transmission efficiency. However, host blood source can influence the infection rate and therefore the subsequent blockage rate. 3) Early-phase transmission could drive an epizootic only in naïve, very susceptible host populations and when the flea burden is high. 4) The low CFU numbers typically transmitted in the early phase may “immunize” many individuals of more resistant host species, acting to limit epizootic spread and promote an enzootic state.

## Materials and methods

### Ethics statement

Fleas were from colonies maintained according to a protocol approved by the Institutional Animal Care and Use Committee of the Rocky Mountain Laboratories (RML) [23]. Animal work adhered to the institution’s guidelines for animal use, the guidelines and principles in the United States Public Health Service Policy on Humane Care and Use of Laboratory Animals, and the Guide for the Care and Use of Laboratory Animals and was conducted by certified staff in an Association for Assessment and Accreditation of Laboratory Animal Care (AAALAC) International accredited facility.

### Flea infections

*O*. *montana* fleas were from a laboratory colony originally established at the CDC, Fort Collins [69] and maintained at RML since 2011. Groups of ~300 *O*. *montana* that had not fed for 5 days were infected by allowing them to feed on 5 ml of defibrinated mouse or rat blood containing 5 to 8 × 10^8^ CFU/ml of *Y*. *pestis* KIM6+ with pAGFP1 (Clontech/Takara Bio) a plasmid that encodes the green fluorescent protein and carbenicillin-resistance or with KIM6+ Δ*hmsH* (pAcGFP1) through a Parafilm M membrane stretched across an artificial feeding device, following a previously established standard protocol [23, 70, 71]. The pAcGFP1 plasmid is stably maintained by *Y*. *pestis* in infected fleas for at least 31 days in the absence of antibiotic pressure. After a 1-h feeding period, fleas were individually examined microscopically, and those that took an infectious blood meal (denoted by the presence of fresh red blood in the midgut) were collected; 20 of them were placed at -80°C for later determination of the initial infectious dose and the rest were placed in capsules containing a layer of sawdust and maintained at 21° C, 75% relative humidity [23].

### Evaluation of flea infection, mortality, blockage, and transmission rates after infection

To assess early-phase transmission, 12–48 individual fleas (equal numbers of males and females) were allowed to feed for 1 h on ~300 μl of sterile defibrinated mouse or rat blood (corresponding to the infectious blood meal source), using miniaturized versions of the artificial feeding device, on days 2 to 4 after infection [71]. Each flea was then examined for evidence of feeding and for partial or complete proventricular blockage [24]. Partial blockage is diagnosed by the presence of fresh blood in the esophagus and midgut and complete blockage by the presence of fresh blood only in the esophagus, usually pooled just anterior to the proventriculus [71]. The blood in the individual reservoirs was removed and spread onto blood agar plates containing 100 μg/ml carbenicillin. CFUs were counted after 48 h at 28° C to determine the number of bacteria transmitted. Fleas that fed were individually frozen at -80°C for subsequent bacterial load determinations.

All other fleas that took an infectious blood meal were provided maintenance feeds twice weekly (Monday and Thursday) beginning 2 to 4 days after infection for one month on a neonatal mouse or on sterile defibrinated rat blood in the same feeding system used to infect them. After the 1-h feeding period fleas were individually examined and the feeding rate and the incidence of partial or complete proventricular blockage was recorded. Flea mortality was also recorded throughout the experiment, and samples of 20 fleas were removed at 7, 14, and 30 days after infection and frozen for bacterial load determination. Fleas showing signs of partial or full blockage were removed from the group and stored separately. These partially and completely blocked fleas were permitted to feed individually on a miniaturized artificial feeder every 1 to 3 days, and evidence of feeding, proventricular blockage status, and mortality were determined each time. Blood was collected from the reservoirs corresponding to those fleas that showed evidence of feeding or attempted feeding and plated to determine the number of CFUs transmitted. Transmissions by the same partially or completely blocked flea on multiple days were each considered as separate transmission events. Between-group differences in transmission probability and number of *Y*. *pestis* CFU transmitted were analyzed by chi-square and Mann-Whitney test, respectively (GraphPad Prism 9 software).

To determine infection rates and the average bacterial load per infected flea, samples of 20 fleas that had been collected and stored at -80°C at different times after the infectious blood meal were thawed, surface-sterilized, individually triturated in PBS, and dilutions plated in BHI soft agar overlays for CFU counts (lower limit of detection = 40 CFU/flea) as previously described [70, 71].

### Deterministic host-vector model

We developed a continuous time, deterministic model formulated to reflect the dynamics of infection in the vector population as they relate to progression of disease in reservoir hosts (Fig 5). The model is based on Bailey’s single host-single vector and Ross-Macdonald models [72, 73] and uses ordinary differential equations to describe the flow of individual vertebrate hosts and vectors through different infection categories (S1 Text). Our model is focused on understanding the relative contribution of the three transmission-competent states of infection in fleas to the dynamics of infection in a host population. Thus, we did not include a birth rate for either flea or rodent compartments.

The flea vector compartment follows a susceptible-infected (SI) pattern with the infected state divided into three successive transmission-competent stages: early-phase (I_ep_; here defined as the first four days after the infectious blood meal), partially blocked (I_pb_), and completely blocked (I_b_) [14, 23]. Additionally, we account for the subset of fleas that clear themselves of infection but that may become reinfected. However, there is no final recovery state because the endstage of biofilm development in the flea (complete blockage) is invariably fatal.

Hosts follow a susceptible-exposed-infected-recovered (SEIR) pattern of disease; however, the exposed-recovered progression is disjointed from the development of overt disease. When the number of CFUs transmitted is less than a lethal dose (LD), we expect that a susceptible host will resolve the infection and recover without ever developing the terminal, high-density bacteremia (>10^7^ *Y*. *pestis*/ml of peripheral blood) that is required to reliably infect fleas that feed on it [29, 33, 43]. Thus, transmission by fleas in any of the three transmission-competent stages (I_ep_, I_pb_, I_b_) to a susceptible host can lead to either exposed but never infectious stages (E, R), or to an infectious stage (I) characterized by a fatal bacteremia. We also included a latent stage (L) prior to the infectious stage to account for the time to develop high bacteremia after transmission.

### Transmission parameter values and conditions for model simulations

Model simulations were performed using the programming language R [74] and were based on parameters generated from experimental results reported here or derived from literature sources. To simulate the cumulative transmission by 10 infected fleas feeding simultaneously in an early-phase challenge, the probability of transmission was calculated as 1 - (1- *p*_ep_)^10^ and the probability of transmitting a lethal dose was calculated as 1 - (1- *t*_ep_)^10^, with *p*_ep_ and *t*_ep_ being the probabilities for a single flea bite (Table 2). It was further assumed that an individual blocked flea would (conservatively) make at least two successive feeding attempts, and the *p*_b_ and *t*_b_ values were similarly adjusted to 1 - (1- *p*_b_)^2^ and 1 - (1- *t*_b_)^2^.

Simulation conditions were set for 100 days at 0.3-day time steps to capture acute infection dynamics. The flea population is represented by *O*. *montana*, and host populations with different susceptibilities (LD_100_ = 1, 10 or 100 *Y*. *pestis* CFUs) were considered. Model populations consisted of 10 hosts (1 bacteremic and 9 uninfected) and 50 uninfected fleas. When infected and dead hosts exceeded 50%, we considered the infection scenario an epizootic. Conversely, when more hosts survived than died from infection, we classified the infection as enzootic. See S1 Text for details of the model and R code.

To understand the role of the different modes of flea-borne transmission of *Y*. *pestis* in maintaining enzootic levels of the pathogen versus stimulating epizootic bursts, we evaluated all flea transmission states together and then systematically compared the individual capacity of early-phase transmission to transmission by blocked/partially blocked fleas by artificially setting one or the other transmission probabilities to zero.

### Estimation of flea density required for enzootic and epizootic transmission

We formulated an expression for R_0_ from our model using the next-generation matrix method [42]. See S1 Text for details and R code. Based on our parameter estimates (Tables 2 and 3, and Fig 3), the number of fleas per host (*m*) that would be required for an enzootic (R_0_ = 1) or epizootic (R_0_ ≥ 2) was calculated.

## Supporting information

S1 Text The deterministic host-vector model; calculating R0; and model code. (PDF)

S1 Fig Model output of the dynamics of plague in host populations with different levels of susceptibility. (PDF)

S2 Fig Model output of the dynamics of plague in host populations with different levels of susceptibility using the modified parameters to account for cumulative transmission by simultaneous flea bites. (PDF)

S3 Fig Model output of the dynamics of flea infection, blockage, and mortality in scenarios with different levels of host susceptibility. (PDF)

S4 Fig Model output of the dynamics of flea infection, blockage, and mortality in scenarios with different levels of host susceptibility using the modified parameters to account for cumulative transmission by simultaneous flea bites. (PDF)

## References

[1] Gage KL, Kosoy M. Recent trends in plague ecology. In: Roelle J, Miller B, Godbey J, Biggins D, editors. Recovery of the black-footed ferret, progress and continuing challenges. Fort. Collins, CO: U. S. Geological Survey; 2006. p. 213–31.

[2] Eisen RJ, Gage KL. Adaptive strategies of *Yersinia pestis* to persist during inter-epizootic and epizootic periods. Vet Res. 2009;40(2):1. 18803931 10.1051/vetres:2008039 PMC2695026

[3] Wimsatt J, Biggins DE. A review of plague persistence with special emphasis on fleas. J Vector Borne Dis. 2009;46:85–99. 19502688

[4] Dubyanskiy VM, Yeszhanov AB. Ecology of *Yersinia pestis* and the epidemiology of plague. Adv Exp Med Biol. 2016;918:101–70. 27722862 10.1007/978-94-024-0890-4_5

[5] Zeppelini CG, de Almeida AM, Cordeiro-Estrela P. Zoonoses as ecological entities: a case review of plague. PLoS Negl Trop Dis. 2016;10(10):e0004949. doi: 10.1371/journal.pntd.0004949 27711205 PMC5053604

[6] Keeling MJ, Gilligan CA. Metapopulation dynamics of bubonic plague. Nature. 2000;407:903–6. doi: 10.1038/35038073 11057668

[7] Davis S, Trapman P, Leirs H, Begon M, Heesterbeek JA. The abundance threshold for plague as a critical percolation phenomenon. Nature. 2008;454:634–7. doi: 10.1038/nature07053 18668107

[8] Durham DP, Casman EA. Threshold conditions for the persistence of plague transmission in urban rats. Risk Anal. 2009;29:1655–63. doi: 10.1111/j.1539-6924.2009.01309.x 19878483

[9] Reijniers J, Davis S, Begon M, Heesterbeek JA, Ageyev VS, Leirs H. A curve of thresholds governs plague epizootics in Central Asia. Ecol Lett. 2012;15:554–60. doi: 10.1111/j.1461-0248.2012.01767.x 22449078

[10] Salkeld DJ, Salathe M, Stapp P, Jones JH. Plague outbreaks in prairie dog populations explained by percolation thresholds of alternate host abundance. Proc Natl Acad Sci USA. 2010;107:14247–50. doi: 10.1073/pnas.1002826107 20660742 PMC2922574

[11] Gascuel F, Choisy M, Duplantier JM, Debarre F, Brouat C. Host resistance, population structure and the long-term persistence of bubonic plague: contributions of a modelling approach in the Malagasy focus. PLoS Comput Biol. 2013;9(5):e1003039. doi: 10.1371/journal.pcbi.1003039 23675291 PMC3649974

[12] Andrianaivoarimanana V, Kreppel K, Elissa N, Duplantier JM, Carniel E, Rajerison M, et al. Understanding the persistence of plague foci in Madagascar. PLoS Negl Trop Dis. 2013;7(11):e2382. doi: 10.1371/journal.pntd.0002382 24244760 PMC3820717

[13] Bacot AW. Further notes on the mechanism of the transmission of plague by fleas. J Hygiene Plague Suppl 4. 1915;14:774–6. 20474604 PMC2206743

[14] Hinnebusch BJ, Jarrett CO, Bland DM. "Fleaing" the plague: Adaptations of *Yersinia pestis* to its insect vector that lead to transmission. Annu Rev Microbiol. 2017;71:215–32. doi: 10.1146/annurev-micro-090816-093521 28886687

[15] Bacot AW, Martin CJ. Observations on the mechanism of the transmission of plague by fleas. J Hygiene Plague Suppl 3. 1914;13:423–39. 20474555 PMC2167459

[16] Bland DM, Jarrett CO, Bosio CF, Hinnebusch BJ. Infectious blood source alters early foregut infection and regurgitative transmission of *Yersinia pestis* by rodent fleas. PLoS Pathog. 2018;14(1):e1006859. 29357385 10.1371/journal.ppat.1006859 PMC5794196

[17] Vetter SM, Eisen RJ, Schotthoefer AM, Montenieri JA, Holmes JL, Bobrov AG, et al. Biofilm formation is not required for early-phase transmission of *Yersinia pestis*. Microbiology. 2010;156:2216–25. 20395271 10.1099/mic.0.037952-0 PMC3068684

[18] Dewitte A, Bouvenot T, Pierre F, Ricard I, Pradel E, Barois N, et al. A refined model of how *Yersinia pestis* produces a transmissible infection in its flea vector. PLoS Pathog. 2020;16(4):e1008440. doi: 10.1371/journal.ppat.1008440 32294143 PMC7185726

[19] Eisen RJ, Gage KL. Transmission of flea-borne zoonotic agents. Ann Rev Entomol. 2012;57:61–82. doi: 10.1146/annurev-ento-120710-100717 21888520

[20] Eisen RJ, Bearden SW, Wilder AP, Montenieri JA, Antolin MF, Gage KL. Early-phase transmission of *Yersinia pestis* by unblocked fleas as a mechanism explaining rapidly spreading plague epizootics. Proc Natl Acad Sci USA. 2006;103:15380–5. 17032761 10.1073/pnas.0606831103 PMC1592641

[21] Eisen RJ, Dennis DT, Gage KL. The role of early-phase transmission in the spread of *Yersinia pestis*. J Med Entomol. 2015;52:1183–92. 26336267 10.1093/jme/tjv128 PMC4636957

[22] Eisen RJ, Eisen L, Gage KL. Studies of vector competency and efficiency of North American fleas for *Yersinia pestis*: state of the field and future research needs. J Med Entomol. 2009;46:737–44. 19645275 10.1603/033.046.0403

[23] Hinnebusch BJ, Bland DM, Bosio CF, Jarrett CO. Comparative ability of *Oropsylla montana* and *Xenopsylla cheopis* fleas to transmit *Yersinia pestis* by two different mechanisms. PLoS Negl Trop Dis. 2017;11(1):e0005276. doi: 10.1371/journal.pntd.0005276 28081130 PMC5230758

[24] Hinnebusch BJ, Perry RD, Schwan TG. Role of the *Yersinia pestis* hemin storage (*hms*) locus in the transmission of plague by fleas. Science. 1996;273:367–70. doi: 10.1126/science.273.5273.367 8662526

[25] Williams SK, Schotthoefer AM, Montenieri JA, Holmes JL, Vetter SM, Gage KL, et al. Effects of low-temperature flea maintenance on the transmission of *Yersinia pestis* by *Oropsylla montana*. Vector Borne Zoonotic Dis. 2013;13:468–78. doi: 10.1089/vbz.2012.1017 23590319

[26] Johnson TL, Hinnebusch BJ, Boegler KA, Graham CB, MacMillan K, Montenieri JA, et al. Yersinia murine toxin is not required for early-phase transmission of *Yersinia pestis* by *Oropsylla montana* (Siphonaptera: Ceratophyllidae) or *Xenopsylla cheopis* (Siphonaptera: Pulicidae). Microbiology. 2014;160:2517–25. doi: 10.1099/mic.0.082123-0 25187626 PMC4612360

[27] Bosio CF, Jarrett CO, Scott DP, Fintzi J, Hinnebusch BJ. Comparison of the transmission efficiency and plague progression dynamics associated with two mechanisms by which fleas transmit *Yersinia pestis*. PLoS Pathog. 2020;16(12):e1009092. doi: 10.1371/journal.ppat.1009092 33284863 PMC7746306

[28] Burroughs AL. Sylvatic plague studies. The vector efficiency of nine species of fleas compared with *Xenopsylla cheopis*. J Hygiene. 1947;45:371–96. 20475778 10.1017/s0022172400014042 PMC2234840

[29] Lorange EA, Race BL, Sebbane F, Hinnebusch BJ. Poor vector competence of fleas and the evolution of hypervirulence in *Yersinia pestis*. J Inf Dis. 2005;191:1907–12. 15871125 10.1086/429931

[30] Eisen RJ, Vetter SM, Holmes JL, Bearden SW, Montenieri JA, Gage KL. Source of host blood affects prevalence of infection and bacterial loads of *Yersinia pestis* in fleas. J Med Entomol. 2008;45:933–8. 18826038 10.1603/0022-2585(2008)45[933:sohbap]2.0.co;2

[31] Burroughs AL. Sylvatic plague studies X. Survival of rodent fleas in the laboratory. Parasitology. 1953;43:35–48. 13046890 10.1017/s0031182000018321

[32] Murphy K, Travers P, Walport M, Janeway C. Janeway’s Immunobiology. 8th ed. New York: Garland Science; 2012.

[33] Engelthaler DM, Hinnebusch BJ, Rittner CM, Gage KL. Quantitative competitive PCR as a technique for exploring flea-*Yersina pestis* dynamics. Am J Trop Med Hyg. 2000;62:552–60. 11289663 10.4269/ajtmh.2000.62.552

[34] Jarrett CO, Sebbane F, Adamovicz JJ, Andrews GP, Hinnebusch BJ. Flea-borne transmission model to evaluate vaccine efficacy against naturally acquired bubonic plague. Infect Immun. 2004;72:2052–6. doi: 10.1128/IAI.72.4.2052-2056.2004 15039326 PMC375218

[35] Sebbane F, Jarrett CO, Gardner D, Long D, Hinnebusch BJ. Role of the *Yersinia pestis* plasminogen activator in the incidence of distinct septicemic and bubonic forms of flea-borne plague. Proc Natl Acad Sci USA. 2006;103:5526–30. 16567636 10.1073/pnas.0509544103 PMC1414629

[36] Sebbane F, Jarrett C, Gardner D, Long D, Hinnebusch BJ. The *Yersinia pestis caf1M1A1* fimbrial capsule operon promotes transmission by flea bite in a mouse model of bubonic plague. Infect Immun. 2009;77:1222–9. 19103769 10.1128/IAI.00950-08 PMC2643634

[37] Sebbane F, Jarrett C, Gardner D, Long D, Hinnebusch BJ. Role of the *Yersinia pestis* yersiniabactin iron acquisition system in the incidence of flea-borne plague. PloS One. 2010;5(12):e14379. doi: 10.1371/journal.pone.0014379 21179420 PMC3003698

[38] Bosio CF, Viall AK, Jarrett CO, Gardner D, Rood MP, Hinnebusch BJ. Evaluation of the murine immune response to *Xenopsylla cheopis* flea saliva and its effect on transmission of *Yersinia pestis*. PLoS Negl Trop Dis. 2014;8(9):e3196. doi: 10.1371/journal.pntd.0003196 25255317 PMC4177749

[39] Dobay A, Pilo P, Lindholm AK, Origgi F, Bagheri HC, Konig B. Dynamics of a tularemia outbreak in a closely monitored free-roaming population of wild house mice. PloS One. 2015;10(11):e0141103. doi: 10.1371/journal.pone.0141103 26536232 PMC4633114

[40] Eisen RJ, Wilder AP, Bearden SW, Montenieri JA, Gage KL. Early-phase transmission of *Yersinia pestis* by unblocked *Xenopsylla cheopis* (Siphonaptera: Pulicidae) is as efficient as transmission by blocked fleas. J Med Entomol. 2007;44:678–82. doi: 10.1603/0022-2585(2007)44[678:etoypb]2.0.co;2 17695025

[41] Wilder AP, Eisen RJ, Bearden SW, Montenieri JA, Tripp DW, Brinkerhoff RJ, et al. Transmission efficiency of two flea species (*Oropsylla tuberculata cynomuris* and *Oropsylla hirsuta*) involved in plague epizootics among prairie dogs. EcoHealth. 2008;5:205–12. doi: 10.1007/s10393-008-0165-1 18787922

[42] Diekmann O, Heesterbeek JA, Roberts MG. The construction of next-generation matrices for compartmental epidemic models. J R Soc Interface. 2010;7(47):873–85. Epub 2009/11/07. doi: 10.1098/rsif.2009.0386 ; PubMed Central PMCID: PMC2871801. 19892718 PMC2871801

[43] Boegler KA, Graham CB, Johnson TL, Montenieri JA, Eisen RJ. Infection prevalence, bacterial loads, and transmission efficiency in *Oropsylla montana* (Siphonaptera: Ceratophyllidae) one day after exposure to varying concentrations of *Yersinia pestis* in blood. J Med Entomol. 2016;53:674–80. doi: 10.1093/jme/tjw004 26843450 PMC6555412

[44] Eskey CR, Haas VH. Plague in the western part of the United States. Washington, D.C.: U.S. Public Health Service; 1940.

[45] Webb CT, Brooks CP, Gage KL, Antolin MF. Classic flea-borne transmission does not drive plague epizootics in prairie dogs. Proc Natl Acad Sci USA. 2006;103:6236–41. doi: 10.1073/pnas.0510090103 16603630 PMC1434514

[46] Wilder AP, Eisen RJ, Bearden SW, Montenieri JA, Gage KL, Antolin MF. *Oropsylla hirsuta* (Siphonaptera: Ceratophyllidae) can support plague epizootics in black-tailed prairie dogs (*Cynomys ludovicianus*) by early-phase transmission of *Yersinia pestis*. Vector Borne Zoonotic Dis. 2008;8:359–67. doi: 10.1089/vbz.2007.0181 18454591

[47] Miarinjara A, Eads DA, Bland DM, Matchett MR, Biggins DE, Hinnebusch BJ. Reevaluation of the role of blocked *Oropsylla hirsuta* prairie dog fleas (Siphonaptera: Ceratophyllidae) in *Yersinia pestis* transmission. J Med Entomol. 2022;59:1053–9. 35380675 10.1093/jme/tjac021 PMC9113170

[48] Kartman L, Prince FM. Studies on *Pasteurella pestis* in fleas. V. The experimental plague-vector efficiency of wild rodent fleas compared with *Xenopsylla cheopis*, together with observations on the influence of temperature. Am J Trop Med Hyg. 1956;5:1058–70. 13381882 10.4269/ajtmh.1956.5.1058

[49] Douglas JR, Wheeler CM. Sylvatic plague studies. II. The fate of *Pasteurella pestis* in the flea. J Inf Dis. 1943;72:18–30.

[50] Wheeler CM, Douglas JR. Sylvatic plague studies V. The determination of vector efficiency. J Inf Dis. 1945;77:1–12.

[51] Lemon A, Cherzan N, Vadyvaloo V. Influence of temperature on development of *Yersinia pestis* foregut blockage in *Xenopsylla cheopis* (Siphonaptera: Pulicidae) and *Oropsylla montana* (Siphonaptera: Ceratophyllidae). J Med Entomol. 2020;57:1997–2007. doi: 10.1093/jme/tjaa113 32533162

[52] Eisen RJ, Lowell JL, Montenieri JA, Bearden SW, Gage KL. Temporal dynamics of early-phase transmission of *Yersinia pestis* by unblocked fleas: secondary infectious feeds prolong efficient transmission by *Oropsylla montana* (Siphonaptera: Ceratophyllidae). J Med Entomol. 2007;44:672–7. doi: 10.1603/0022-2585(2007)44[672:tdoeto]2.0.co;2 17695024

[53] Eisen RJ, Holmes JL, Schotthoefer AM, Vetter SM, Montenieri JA, Gage KL. Demonstration of early-phase transmission of *Yersinia pestis* by the mouse flea, *Aetheca wagneri* (Siphonaptera: Ceratophylidae), and implications for the role of deer mice as enzootic reservoirs. J Med Entomol. 2008;45:1160–4. doi: 10.1603/0022-2585(2008)45[1160:doetoy]2.0.co;2 19058643

[54] Buhnerkempe MG, Eisen RJ, Goodell B, Gage KL, Antolin MF, Webb CT. Transmission shifts underlie variability in population responses to *Yersinia pestis* infection. PloS One. 2011;6(7):e22498. doi: 10.1371/journal.pone.0022498 21799873 PMC3143141

[55] Kartman L, Quan SF, McManus AG. Studies on *Pasteurella pestis* in fleas. IV. Experimental blocking of *Xenopsylla vexabilis hawaiiensis* and *Xenopsylla cheopis* with an avirulent strain. Exp Parasitol. 1956;5:435–40. 13365622 10.1016/s0014-4894(56)80003-9

[56] Kartman L, Prince FM, Quan SF. Studies on *Pasteurella pestis* in fleas VII. The plague-vector efficiency of *Hystrichopsylla linsdalei* compared with *Xenopsylla cheopis* under experimental conditions. Am J Trop Med Hyg. 1958;7:317–22. 13533739 10.4269/ajtmh.1958.7.317

[57] Hinnebusch BJ, Rudolph AE, Cherepanov P, Dixon JE, Schwan TG, Forsberg Å. Role of Yersinia murine toxin in survival of *Yersinia pestis* in the midgut of the flea vector. Science. 2002;296:733–5. 11976454 10.1126/science.1069972

[58] Lemon A, Sagawa J, Gravelle K, Vadyvaloo V. Biovar-related differences apparent in the flea foregut colonization phenotype of distinct *Yersinia pestis* strains do not impact transmission efficiency. Parasit Vectors. 2020;13(1):335. doi: 10.1186/s13071-020-04207-x 32611387 PMC7329463

[59] Kartman L, Prince FM, Quan SF, Stark HE. New knowledge on the ecology of sylvatic plague. Ann N Y Acad Sci. 1958;70:668–711. doi: 10.1111/j.1749-6632.1958.tb35421.x 13559927

[60] Pollitzer R. Plague. Geneva: World Health Organization; 1954.

[61] Lillard JW, Bearden SW, Fetherston JD, Perry RD. The haemin storage (Hms+) phenotype of *Yersinia pestis* is not essential for the pathogenesis of bubonic plague in mammals. Microbiology. 1999;145(:197–209. doi: 10.1099/13500872-145-1-197 10206699

[62] Sun YC, Jarrett CO, Bosio CF, Hinnebusch BJ. Retracing the evolutionary path that led to flea-borne transmission of *Yersinia pestis*. Cell Host Microbe. 2014;15:578–86. 24832452 10.1016/j.chom.2014.04.003 PMC4084870

[63] Gandon S, Heitzmann L, Sebbane F. To block or not to block: The adaptive manipulation of plague transmission. Evol Lett. 2019;3:152–61. doi: 10.1002/evl3.111 31161047 PMC6541909

[64] McCoy GW. Studies upon plague in ground squirrels. Publ Hlth Bull. 1911;43:3–51.

[65] Williams JE, Moussa MA, Cavanaugh DC. Experimental plague in the California ground squirrel. J Infect Dis. 1979;140:618–21. doi: 10.1093/infdis/140.4.618 512421

[66] Quan TJ, Barnes AM, Carter LG, Tsuchiya KR. Experimental plague in rock squirrels, *Spermophilus variegatus* (Erxleben). J Wildl Dis. 1985;21:205–10. doi: 10.7589/0090-3558-21.3.205 4032620

[67] Pulliam JRC, Dushoff JG, Levin SA, Dobson AP. Epidemic enhancement in partially immune populations. PloS One. 2007;2(1) e165. doi: 10.1371/journal.pone.0000165 17225866 PMC1769520

[68] Tedrow RE, Zimmerman PA, Abbott KC. Multiple blood feeding: a force multiplier for transmission. Trends Parasitol. 2019;35:949–52. doi: 10.1016/j.pt.2019.08.004 31585840

[69] Engelthaler DM, Gage KL, Montenieri JA, Chu M, Carter LG. PCR detection of *Yersinia pestis* in fleas: comparison with mouse inoculation. J Clin Microbiol. 1999;37:1980–4. 10325359 10.1128/jcm.37.6.1980-1984.1999 PMC85002

[70] Bland DM, Hinnebusch BJ. Feeding behavior modulates biofilm-mediated transmission of *Yersinia pestis* by the cat flea, *Ctenocephalides felis*. PLoS Negl Trop Dis. 2016;10(2):e0004413. doi: 10.1371/journal.pntd.0004413 26829486 PMC4734780

[71] Bland DM, Brown LD, Jarrett CO, Hinnebusch BJ, Macaluso KR. Methods in Flea Research [Internet]. BEI Resources. 2017. Available from: https://www.beiresources.org/Portals/2/Methods%20in%20Flea%20Research%20v2.pdf.

[72] Bailey NTJ. The Biomathematics of Malaria. London: Charles Griffin & Co. Ltd.; 1982.

[73] Smith DL, Battle KE, Hay SI, Barker CM, Scott TW, McKenzie FE. Ross, Macdonald, and a theory for the dynamics and control of mosquito-transmitted pathogens. PLoS Pathog. 2012;8(4):e1002588. doi: 10.1371/journal.ppat.1002588 22496640 PMC3320609

[74] R Core Team. R: A language and environment for statistical computing. Vienna, Austria: R Foundation for Statistical Computing; 2019.

