# Supplementary material for: A Role for Early-Phase Transmission in the Enzootic Maintenance of Plague
Source: PLoS Pathog. 2022 Dec 15;18(12):e1010996. doi: 10.1371/journal.ppat.1010996 (PMC9754260; doi:10.1371/journal.ppat.1010996)
Supplement: S1 Text — (PDF) [file ppat.1010996.s001.pdf]

Supporting Information

The Deterministic host-vector model

The flea sub-model is described as follows:

$$\frac{dU}{dt} = -IUb\alpha/H + I_{ep}(\lambda_B + \lambda_C) - U\mu_F \quad [1]$$

$$\frac{dI_{ep}}{dt} = IUb\alpha/H - I_{ep}(\lambda_A + \lambda_B + \lambda_C + \mu_F) \quad [2]$$

$$\frac{dI_{pb}}{dt} = I_{ep}\lambda_A - I_{pb}(\tau + \mu_{pb}) \quad [3]$$

$$\frac{dI_b}{dt} = \mathrm{I}_{pb}\tau - \mu_b I_b \quad [4]$$

Uninfected fleas (U) enter the early-phase infected state (I_{ep}) when they feed on a bacteremic host (I), at an incidence determined by their biting frequency (b), the number of fleas per host (H) and the proportion of fleas productively infected after the infectious blood meal (α, which is largely dependent on the bacteremia level) [1, 2]. These types of models assume homogenous mixing, where all individuals are weakly interacting. Individuals are able to return to the uninfected state as they clear infection during early-phase (λ_B) [Eq. 1]. From early-phase, fleas either progress to partial blockage (I_{pb}) at rate λ_A, clear the infection (λ_B), or maintain the infection after the early phase without developing a biofilm-mediated partial or complete blockage (λ_C; these fleas essentially return to the U state, because if they later feed on another infectious host they would reenter the pipeline at the early-phase stage) [Eq. 2, 3]. Additionally, fleas in the uninfected and early-phase classes may die at a natural mortality rate of μ_F. From the partially blocked class (I_{pb}), fleas either progress to full blockage at rate τ or die from complications associated with partial biofilm development (μ_{pb}) [Eq. 4]. Once fleas reach the fully blocked state (I_b), the infection is terminal and individuals die from blockage-induced starvation (μ_b).

The rodent host sub-model is detailed below:

$$\frac{dS}{dt} = -S(b\mathrm{I}_{ep}\, p_{ep} + b\mathrm{I}_{pb}\, p_{pb} + b_b\mathrm{I}_b\, p_b)/H - \mu_R S \quad [5]$$

$$\frac{dL}{dt} = S\big(b\mathrm{I}_{ep}p_{ep}t_{ep} + b\mathrm{I}_{pb}p_{pb}t_{pb} + b_b\mathrm{I}_b p_b t_b\big)/H - \sigma\mathrm{L} - \mu_R\mathrm{L} \quad [6]$$

$$\frac{dI}{dt} = \sigma\mathrm{L} - I(\varepsilon + \mu_R) \quad [7]$$

$$\frac{dE}{dt} = S\big[b\mathrm{I}_{ep}p_{ep}(1 - t_{ep}) + b\mathrm{I}_{pb}p_{pb}(1 - t_{pb}) + b_b\mathrm{I}_b p_b(1 - t_b)\big]/H - E(\mu_R + \gamma) \quad [8]$$

$$\frac{dR}{dt} = \gamma E - \mu_R R \quad [9]$$

The force of infection to susceptible hosts from fleas in any of the three transmission-competent stages (I_{ep}, I_{pb}, I_b) is dependent on their biting frequency (b for uninfected, early-phase, and partially blocked fleas; b_b for blocked fleas) multiplied by the proportion of bites that actually result in transmission (p_{ep}, p_{pb}, p_b, for the transmission probabilities of early-phase, partially blocked, and fully blocked fleas, respectively) and the total number of hosts (H) [Eq. 5]. Only the proportion of these positive bites in which a lethal dose is transmitted (t, the transmission efficiency coefficient) lead to the latent (L) and infectious, bacteremic (I) host stages, whereas the remaining proportion of bites in which the number of *Y. pestis* transmitted is below this threshold (1-t) result in noninfectious, recovered hosts (E and R stages) [Eq. 6-9].

Calculating R_0

R_0 can be calculated as the dominant eigenvalue of the next-generation matrix (NGM) at the disease-free equilibrium. The NGM is calculated by multiplying the transmission matrix, $\boldsymbol{T}$, by the negative inverse of the transition matrix, $-\boldsymbol{\Sigma}^{-1}$. $\boldsymbol{T}$ and $\boldsymbol{\Sigma}$ are Jacobians - matrices of partial derivatives of the relevant equations at their disease-free equilibrium with respect to each of the infected classes, only including the terms that describe new infections (transmission matrix, $\boldsymbol{T}$) or transition between infected classes (transition matrix, $\boldsymbol{\Sigma}$). [3].

The transmission matrix, $\boldsymbol{T}$, for our model is

$$\boldsymbol{T} = \begin{pmatrix} 0 & 0 & b \cdot p_{ep} \cdot t_{ep} & b \cdot p_{pb} \cdot t_{pb} & b_1 \cdot p_b \cdot t_b \\ 0 & 0 & 0 & 0 & 0 \\ 0 & U \cdot b \cdot \alpha / H & 0 & 0 & 0 \\ 0 & 0 & 0 & 0 & 0 \\ 0 & 0 & 0 & 0 & 0 \end{pmatrix}$$

The transition matrix, $\boldsymbol{\Sigma}$, for our model is

$$\boldsymbol{\Sigma} = \begin{pmatrix} -(\sigma + \mu_R) & 0 & 0 & 0 & 0 \\ \sigma & -(\mu_R + \epsilon) & 0 & 0 & 0 \\ 0 & 0 & -(\lambda_A + \lambda_B + \lambda_c + \mu_F) & 0 & 0 \\ 0 & 0 & \lambda_A & -(\tau + \mu_{pb}) & 0 \\ 0 & 0 & 0 & \tau & -\mu_b \end{pmatrix}$$

Using the rSymPy package we calculated R_0 as the dominant eigenvalue of the next-generation matrix (NGM). Here, m, is the vector to host ratio: U/H.

$$R_0 = \left(-\alpha \cdot m \cdot p_{ep} \cdot \sigma \cdot t_{ep} \cdot \frac{b^2}{\left((-\epsilon - \mu_R) \cdot (-\mu_R - \sigma) \cdot (-\lambda_A - \lambda_B - \lambda_C - \mu_F)\right)} + \alpha \cdot \lambda_A \cdot m \cdot p_{pb} \cdot \sigma \cdot t_{pb} \cdot \frac{b^2}{\left((-\epsilon - \mu_R) \cdot (-\mu_R - \sigma) \cdot (-\mu_{pb} - \tau) \cdot (-\lambda_A - \lambda_B - \lambda_C - \mu_f)\right)} + \alpha \cdot b \cdot b_b \cdot \lambda_A \cdot m \cdot p_b \cdot \sigma \cdot \tau \cdot \frac{t_b}{\left(\mu_b \cdot (-\epsilon - \mu_R) \cdot (-\mu_R - \sigma) \cdot (-\mu_{pb} - \tau) \cdot (-\lambda_A - \lambda_B - \lambda_C - \mu_f)\right)}\right)^{\frac{1}{2}}$$

Model Code

```
SIR.model.fleasperhost=function(t,x,params){
  b=params["b"]       #biting rate of fleas
  bb=params["bb"]     #biting rate of blocked fleas
  tep=params["tep"]   #proportion of INFECTIOUS bites from early-phase fleas
  tpb=params["tpb"]   #proportion of INFECTIOUS bites from partially blocked
fleas
  tb=params["tb"]    #proportion of INFECTIOUS bites from fully blocked fleas
  mu=params["mu"]    #natural mortality rate of rodent (uninfected)
  pep=params["pep"]  #proportion of ep fleas that transmit
  ppb=params["ppb"]  #proportion of partially blocked fleas that transmit
  pb=params["pb"]    #proportion of fully blocked fleas that transmit
  gamma=params["gamma"] #recovery rate in rodent from low-dose flea infection
  epsilon=params["epsilon"]   #disease induced mortality rate in rodent from
high-dose flea infection
  sigma=params["sigma"]  #rate to become infectious from latent class

  alpha=params["alpha"]  #proportion of fleas infected from host
  muf=params["muf"]      #natural mortality of flea
  mupb=params["mupb"]    #mortality of partially blocked flea
  mub=params["mub"]      #mortality of blocked flea

  lambdaA=params["lambdaA"]  #rate of developing partial blockage from early
phase
  lambdaB=params["lambdaB"] #rate of clearing infection in early phase (back
to uninfected)
  lambdaC=params["lambdaC"] #rate of leaving early phase (still infected but
not enough to block)
  tau=params["tau"]         #rate of developing full blockage

  S=x[1]  # susceptible rodents
  L=x[2]  # rodents latently infected (pre-infectious)
  I=x[3]  # rodents infected (bacteremic)
  E=x[4]  # rodents exposed (non-infectious)
  R=x[5]  # rodents recovered and immune
```

```
  dr=x[6] # all rodents that died
  Id=x[7] # rodents that died of infection

  U=x[8]    # uninfected fleas
  Iep=x[9]  # early phase fleas
  Ipb=x[10] # partially blocked fleas
  Ib=x[11]  # fully blocked fleas
  df=x[12]  # dead fleas

  H  = S+R+E+L+I # total number of rodent hosts

  dS = -S*(b*Iep*pep + b*Ipb*ppb + bb*Ib*pb)/H - mu*S
  dL = S*(b*Iep*pep*tep + b*Ipb*ppb*tpb + bb*Ib*pb*tb)/H - sigma*L - mu*L
  dI = sigma*L - I*(mu+epsilon)
  dE = S*(b*Iep*pep*(1-tep) + b*Ipb*ppb*(1-tpb) + bb*Ib*pb*(1-tb))/H -
        E*(mu+gamma)
  dR = gamma*E - R*mu
  dr = mu*H + epsilon*I
  Id = epsilon*I

  dU=-I*(U*b*alpha)/H + Iep*lambdaB + Iep*lambdaC - U*muf
  dIep=I*U*b*alpha/H - (Iep*lambdaB + Iep*lambdaC + Iep*lambdaA + Iep*muf)
  dIpb=Iep*lambdaA - Ipb*(tau+mupb)
  dIb=tau*Ipb - mub*Ib
  df=muf*(U+Iep) + mupb*Ipb + mub*Ib
  cumb= tau*Ipb


  list(c(dS,dL,dI,dE,dR,dr,Id, dU,dIep,dIpb,dIb,df,cumb))

}
```

Function to Run the Model Comparing Several Scenarios

```
print.plague.SIRmodel <- function(
      model = SIR.model.fleasperhost,
      params, # list of vectors of parameters with labels for each scenario
      xstart, # starting values
      T, # number of days to simulate
      plot=TRUE, # if TRUE, save pdf of plots in dir
      plot.name=NULL){ #optional name for plot pdf file

  times=seq(0,T,by=0.3)                   #time steps to output

  out <- list()
  for(i in 1:length(params)){
    out [[i]] <- as.data.frame(lsoda(xstart, times, model, params[[i]],
                                     hmax = 0.001))  #run the model
```

```
  }
  names(out) <- names(params)

  if(plot==TRUE){
    if(length(plot.name)==0){
      plot.name="plague.plot"
    }
    pdf(plot.name, onefile = TRUE, paper = "special", height = 7, width = 11)
    par(mfrow=c(length(params)/3,3))

    for(i in 1:length(params)){
      dat <- as.data.frame(out[[i]])
      plot(dat$time,dat$I,ylab="Abundance",xlab="Time (days)",type="l",col="r
ed",lwd=2,ylim=c(0,sum(dat[1,2:8])),lty=1)
      lines(dat$time,dat$E,col="blue",lwd=2,lty=1)
      lines(dat$time,dat$S,col="forestgreen",lwd=2,lty=1)
      lines(dat$time,dat$L,col="red",lwd=2,lty=5)
      lines(dat$time,dat$R,col="blue",lwd=2,lty=5)
      lines(dat$time,dat$Id,col="black",lwd=2,lty=1)
      legend("topright",c("Susceptible", "Latent", "Infectious","Exposed",
              "Recovered", "Infected-dead"),col=c("forestgreen","red", "red",
              "blue", "blue", "black"),bty="n",lty=c(1, 5, 1, 1, 5, 1),lwd=2,
              seg.len=2.0,x.intersp =0.5, y.intersp =1)
      title(main=names(params)[i])
    }

    par(mfrow=c(length(params)/3,3))

    for(i in 1:length(params)){
      dat <- as.data.frame(out[[i]])
      plot(dat$time,dat$U,ylab="Abundance",xlab="Time (days)",type="l",col="b
lue",lwd=2,ylim=c(0,sum(dat[1,9:13])),lty=1)
      lines(dat$time,dat$Iep,col="forestgreen",lwd=2,lty=1)
      lines(dat$time,dat$Ipb,col="magenta",lwd=2,lty=1)
      lines(dat$time,dat$Ib,col="red",lwd=2,lty=1)
      lines(dat$time,dat$df,col="black",lwd=2,lty=5)
      lines(dat$time,dat$cumb,col="orange",lwd=2,lty=5)
      legend("topright",c("U", "Iep", "Ipb","Ib", "dead","cumBlocked"),
             col=c("blue","forestgreen","magenta", "red", "black","orange"),
             bty="n",lty=c(1, 1, 1, 1, 5, 5),lwd=2,seg.len=2.0,x.intersp =0.5
,
             y.intersp =1)
      title(main=names(params)[i])
    }

    dev.off()
  }

  ## table of summaries
```

```
  summary.table <- data.frame(scenario=names(params),R0=rep(NA,length(params)
),
                        Idead=rep(NA,length(params)),Recovered=rep(NA,
                        length(params)),cumblocked=rep(NA,length(params)))

  for(i in 1:length(out)){
    dat <- as.data.frame(out[[i]])
    summary.table$R0[i] = R0.function(params[[i]], m=xstart[8]/sum(xstart[1:7
]))
    summary.table$Idead[i] = dat$Id[dim(dat)[1]]
    summary.table$Recovered[i] = dat$R[dim(dat)[1]]
    summary.table$cumblocked[i] = dat$cumb[dim(dat)[1]]

  }

  print(summary.table)
  return(out)
}
```

Code to Run the Model Using Comparison Function

```
library(deSolve)

#############################################
### Baseline parameter estimates
#############################################

params.mouse_1CFU <- c(alpha=1,lambdaA=0.035,lambdaB=0.20,lambdaC=0.07,b=0.4,
                       bb=2,tau=0.39, muf=0.02, mupb=0.14,mub=0.20,
                       pep=0.18,ppb=0.11,pb=0.5,tep=1,tpb=1,tb=1,
                       mu=0.002,sigma=0.25,gamma=0.07,epsilon=0.5)
params.rat_1CFU <- c(alpha=1,lambdaA=0.04,lambdaB=0.02,lambdaC=0.06,b=0.4,
                       bb=2,tau=0.48, muf=0.02, mupb=0.13,mub=0.26,
                       pep=0.14,ppb=0.10,pb=0.67,tep=1,tpb=1,tb=1,
                       mu=0.002,sigma=0.25,gamma=0.07,epsilon=0.5)
params.mouse_10CFU <- c(alpha=1,lambdaA=0.035,lambdaB=0.20,lambdaC=0.07,
                        b=0.4,bb=2,tau=0.39,muf=0.02,mupb=0.14,mub=0.20,
                        pep=0.18,ppb=0.11,pb=0.5,tep=0.00,tpb=0.5,tb=0.8,
                        mu=0.002,sigma=0.25,gamma=0.07,epsilon=0.5)
params.rat_10CFU <- c(alpha=1,lambdaA=0.04,lambdaB=0.02,lambdaC=0.06,b=0.4,
                      bb=2,tau=0.48,muf=0.02,mupb=0.13,mub=0.26,
                      pep=0.14,ppb=0.10,pb=0.67,tep=0.5,tpb=1,tb=0.8,
                      mu=0.002,sigma=0.25,gamma=0.07,epsilon=0.5)
params.mouse_100CFU <- c(alpha=1,lambdaA=0.035,lambdaB=0.20,lambdaC=0.07,
                         b=0.4,bb=2,tau=0.39,muf=0.02,mupb=0.14,mub=0.20,
                         pep=0.18,ppb=0.11,pb=0.5,tep=0.00,tpb=0,tb=0.65,
                         mu=0.002,sigma=0.25,gamma=0.07,epsilon=0.5)
```

```r
params.rat_100CFU <- c(alpha=1,lambdaA=0.04,lambdaB=0.02,lambdaC=0.06,b=0.4,
                       bb=2,tau=0.48,muf=0.02,mupb=0.13,mub=0.26,
                       pep=0.14,ppb=0.10,pb=0.67,tep=0,tpb=1,tb=0.41,
                       mu=0.002,sigma=0.25,gamma=0.07,epsilon=0.5)


##########################################################################
### Now with Early Phase Only
##########################################################################

params.mouse_1CFU.EPTonly <-  params.mouse_1CFU
  params.mouse_1CFU.EPTonly[c("ppb","pb","tpb","tb")]<-0
params.rat_1CFU.EPTonly <- params.rat_1CFU
  params.rat_1CFU.EPTonly[c("ppb","pb","tpb","tb")] <- 0
params.mouse_10CFU.EPTonly <- params.mouse_10CFU
  params.mouse_10CFU.EPTonly[c("ppb","pb","tpb","tb")] <- 0
params.rat_10CFU.EPTonly <- params.rat_10CFU
  params.rat_10CFU.EPTonly[c("ppb","pb","tpb","tb")] <- 0
params.mouse_100CFU.EPTonly <- params.mouse_100CFU
  params.mouse_100CFU.EPTonly[c("ppb","pb","tpb","tb")] <- 0
params.rat_100CFU.EPTonly <- params.rat_100CFU
  params.rat_100CFU.EPTonly[c("ppb","pb","tpb","tb")] <- 0


##########################################################################
### Now with no early phase, only partially and fully blocked
##########################################################################

params.mouse_1CFU.BPBonly <-  params.mouse_1CFU
  params.mouse_1CFU.BPBonly[c("pep","tep")]<-0
params.rat_1CFU.BPBonly <- params.rat_1CFU
  params.rat_1CFU.BPBonly[c("pep","tep")] <- 0
params.mouse_10CFU.BPBonly <- params.mouse_10CFU
  params.mouse_10CFU.BPBonly[c("pep","tep")] <- 0
params.rat_10CFU.BPBonly <- params.rat_10CFU
  params.rat_10CFU.BPBonly[c("pep","tep")] <- 0
params.mouse_100CFU.BPBonly <- params.mouse_100CFU
  params.mouse_100CFU.BPBonly[c("pep","tep")] <- 0
params.rat_100CFU.BPBonly <- params.rat_100CFU
  params.rat_100CFU.BPBonly[c("pep","tep")] <- 0



##################################################################
# Run model using comparison function
##################################################################

params.mouse <- list(params.mouse_1CFU, params.mouse_1CFU.EPTonly,
                     params.mouse_1CFU.BPBonly, params.mouse_10CFU,
```

```
                     params.mouse_10CFU.EPTonly, params.mouse_10CFU.BPBonly,
                     params.mouse_100CFU, params.mouse_100CFU.EPTonly,
                     params.mouse_100CFU.BPBonly)
names(params.mouse) <- c("mouse_1CFU","mouse_1CFU.EPTonly",
                         "mouse_1CFU.BPBonly", "mouse_10CFU",
                         "mouse_10CFU.EPTonly", "mouse_10CFU.BPBonly",
                         "mouse_100CFU", "mouse_100CFU.EPTonly",
                         "mouse_100CFU.BPBonly")

SIRcum.mouse.comparisons <- print.plague.SIRmodel(
  model = SIR.model.fleasperhost,
  params = params.mouse,
  xstart=c(S=9,L=0,I=1,E=0,R=0,dr=0,Id=0,
           U=50,Iep=0,Ipb=0,Ib=0,df=0,cumb=0),
  T=100,
  plot.name="Plague SIR, m=5, mouse comparison.pdf")



params.rat <- list(params.rat_1CFU, params.rat_1CFU.EPTonly,
                   params.rat_1CFU.BPBonly, params.rat_10CFU,
                   params.rat_10CFU.EPTonly, params.rat_10CFU.BPBonly,
                   params.rat_100CFU, params.rat_100CFU.EPTonly,
                   params.rat_100CFU.BPBonly)
names(params.rat) <- c("rat_1CFU","rat_1CFU.EPTonly", "rat_1CFU.BPBonly",
                       "rat_10CFU", "rat_10CFU.EPTonly", "rat_10CFU.BPBonly",
                       "rat_100CFU", "rat_100CFU.EPTonly",
                       "rat_100CFU.BPBonly")

SIR.rat.comparisons <- print.plague.SIRmodel(
  model = SIR.model.fleasperhost,
  params = params.rat,
  xstart=c(S=9,L=0,I=1,E=0,R=0,dr=0,Id=0,
           U=50,Iep=0,Ipb=0,Ib=0,df=0,cumb=0),
  T=100,
  plot.name="Plague SIR, m=5, rat comparison.pdf")
```

Code to Calculate R_0 Using Next-Generation Matrix Method

```
library(rSymPy)

# define variables for the SymPy workspace
b <- Var("b")
bb  <- Var("bb")
S <- Var("S")
H <- Var("H")
tep <- Var("tep")
```

```
pep <- Var("pep")
Iep <- Var("Iep")
tpb <- Var("tpb")
ppb <- Var("ppb")
Ipb <- Var("Ipb")
tb <- Var("tb")
pb <- Var("pb")
Ib <- Var("Ib")
U <- Var("U")
alpha <- Var("alpha")
I <- Var("I")
sigma <- Var("sigma")
mu <- Var("mu")
epsilon <- Var("epsilon")
lambdaA <- Var("lambdaA")
lambdaB <- Var("lambdaB")
lambdaC <- Var("lambdaC")
muf <- Var("muf")
tau <- Var("tau")
mupb <- Var("mupb")
mub <- Var("mub")
m <- Var("m") # vector to host ratio (U/H)

sympy("Ltrans = (b*Iep*pep*tep + b*Ipb*ppb*tpb + bb*Ib*pb*tb)")

sympy("T_L_Iep = diff(Ltrans,Iep,1)")
sympy("T_L_Ipb = diff(Ltrans,Ipb,1)")
sympy("T_L_Ib = diff(Ltrans,Ib,1)")
sympy("T_Iep_I = diff(I*m*b*alpha,I,1)")

# transmission matrix, T
cat(sympy("T = Matrix([[0,0,T_L_Iep,T_L_Ipb,T_L_Ib],
          [0,0,0,0,0],
          [0,T_Iep_I,0,0,0],
          [0,0,0,0,0],
          [0,0,0,0,0]])"),"\n")


sympy("E_L_L = -(sigma+mu)")
sympy("E_I_L = sigma")
sympy("E_I_I = -(mu+epsilon)")
sympy("E_Iep_Iep = -(lambdaA+lambdaB+lambdaC+muf)")
sympy("E_Ipb_Iep = lambdaA")
sympy("E_Ipb_Ipb = -(tau+mupb)")
sympy("E_Ib_Ipb = tau")
sympy("E_Ib_Ib = -mub")

## transition matrix E (Sigma)
cat(sympy("E = Matrix([[E_L_L,0,0,0,0],
```

```
      [E_I_L,E_I_I,0,0,0],
      [0,0,E_Iep_Iep,0,0],
      [0,0,E_Ipb_Iep,E_Ipb_Ipb,0],
      [0,0,0,E_Ib_Ipb,E_Ib_Ib]])"),"\n")


Sym("NegInvE = -E.inv()") # take the negative inverse of E


sympy("NGM = T*NegInvE")
Sym("R0 = NGM.eigenvals()")
```

References

1. Engelthaler DM, Hinnebusch BJ, Rittner CM, Gage KL. Quantitative competitive PCR as a technique for exploring flea-*Yersina pestis* dynamics. Am J Trop Med Hyg. 2000;62:552-60.

2. Lorange EA, Race BL, Sebbane F, Hinnebusch BJ. Poor vector competence of fleas and the evolution of hypervirulence in *Yersinia pestis*. J Inf Dis. 2005;191:1907-12.

3. Diekmann O, Heesterbeek JA, Roberts MG. The construction of next-generation matrices for compartmental epidemic models. J R Soc Interface. 2010;7:873-85.
