# Supplementary material for: A Role for Early-Phase Transmission in the Enzootic Maintenance of Plague
Source: PLoS Pathog. 2022 Dec 15;18(12):e1010996. doi: 10.1371/journal.ppat.1010996 (PMC9754260; doi:10.1371/journal.ppat.1010996)
Supplement: S1 Fig — (PDF) [file ppat.1010996.s002.pdf]

Fig. S1A. Fleas infected using mouse blood

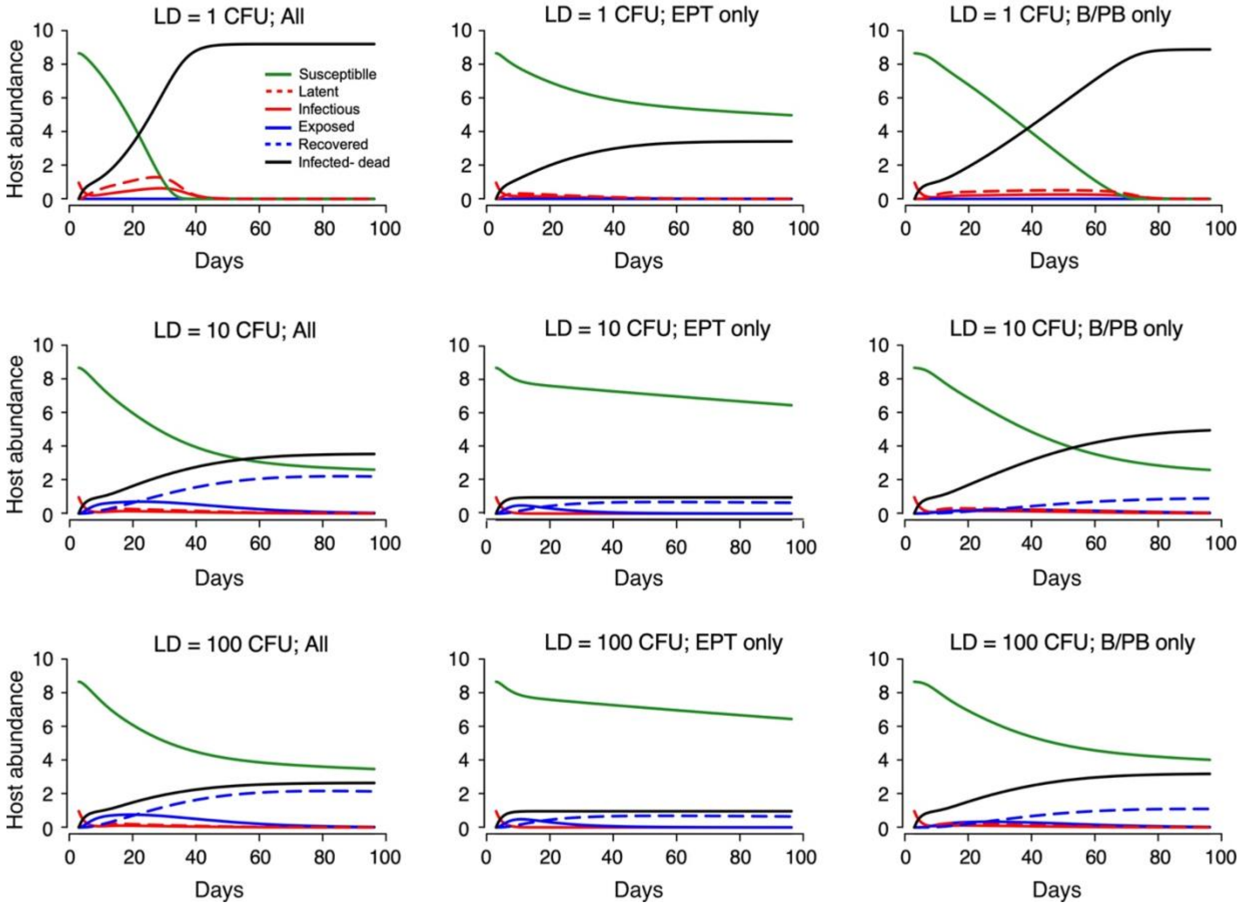

	Host outcome (%)		
Scenario (mouse blood)	Dead	Recovered	Susceptible
1 CFU; All	96	0	0
1 CFU; EPT only	36	0	52
1 CFU; B/PB only	92	0	0
10 CFU; All	36	23	27
10 CFU; EPT only	10	7	67
10 CFU; B/PB only	51	9	27
100 CFU; All	27	22	36
100 CFU; EPT only	10	7	67
100 CFU; B/PB only	33	11	42

Fig. S1B. Fleas infected using rat blood

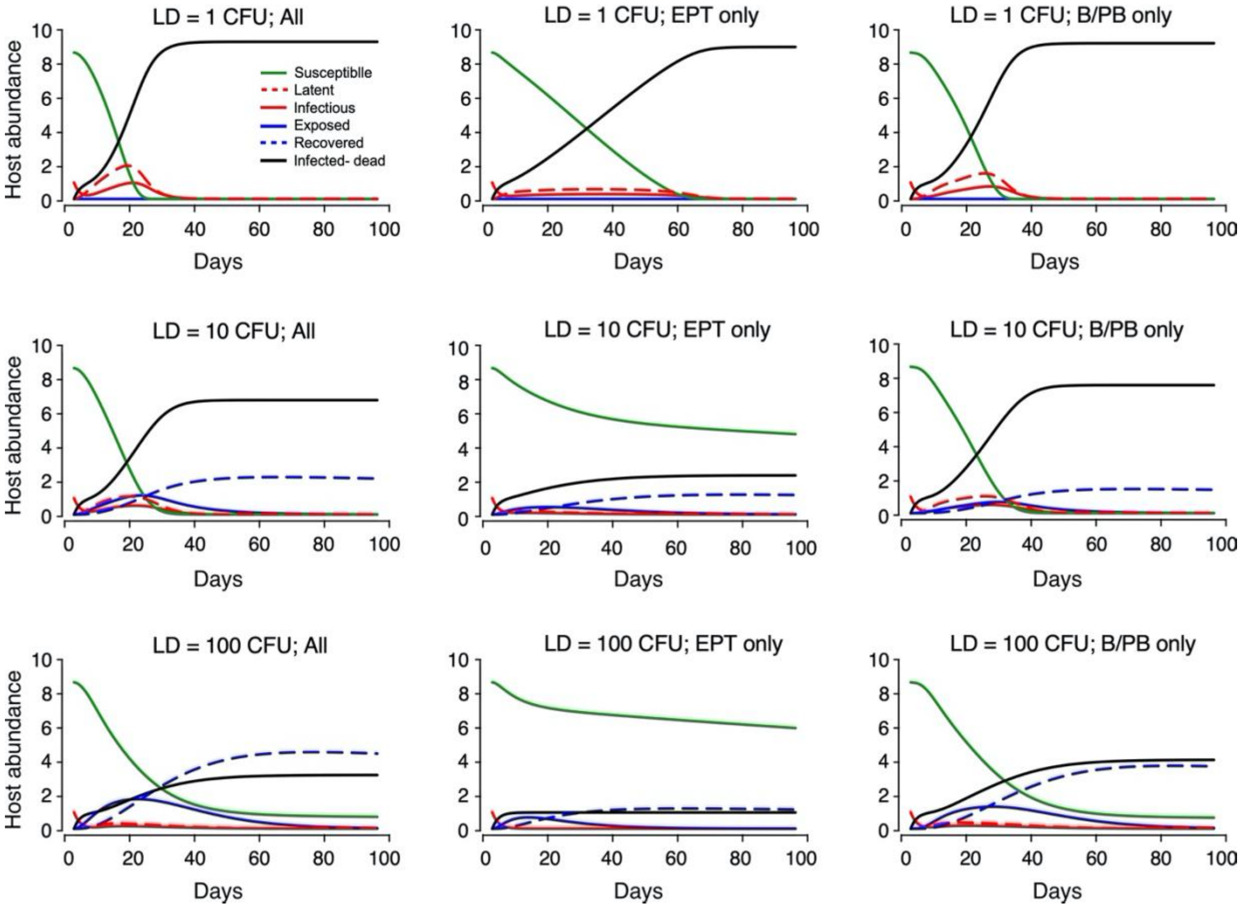

	Host outcome (%)		
Scenario (rat blood)	Dead	Recovered	Susceptible
1 CFU; All	96	0	0
1 CFU; EPT only	93	0	0
1 CFU; B/PB only	96	0	0
10 CFU; All	70	20	0
10 CFU; EPT only	24	7	49
10 CFU; B/PB only	79	14	0
100 CFU; All	33	44	7
100 CFU; EPT only	10	10	62
100 CFU; B/PB only	42	42	7

S1 Fig. Model output of the dynamics of plague in host populations with different levels of susceptibility [lethal dose (LD) of 1, 10, or 100 *Y. pestis* CFU], using the unmodified parameters in Table 2. Separate outcomes produced by fleas infected using mouse blood (**A**) or rat blood (**B**) in which both early-phase transmission and biofilm-dependent transmission by partially and completely blocked fleas are operative (All); or in which only early-phase transmission (EPT only) or only biofilm-dependent transmission (B/PB only) are operative are indicated. All simulations were initiated with 9 susceptible hosts, 1 infected (highly bacteremic) host, and 50 uninfected fleas. The percentage of infected-dead, infected-recovered, and uninfected, still susceptible hosts at the end of the 100-day simulation period and the R_0 values are shown in the Table.
