# Supplementary material for: A Role for Early-Phase Transmission in the Enzootic Maintenance of Plague
Source: PLoS Pathog. 2022 Dec 15;18(12):e1010996. doi: 10.1371/journal.ppat.1010996 (PMC9754260; doi:10.1371/journal.ppat.1010996)
Supplement: S2 Fig — (PDF) [file ppat.1010996.s003.pdf]

Fig. S2A. Fleas infected using mouse blood

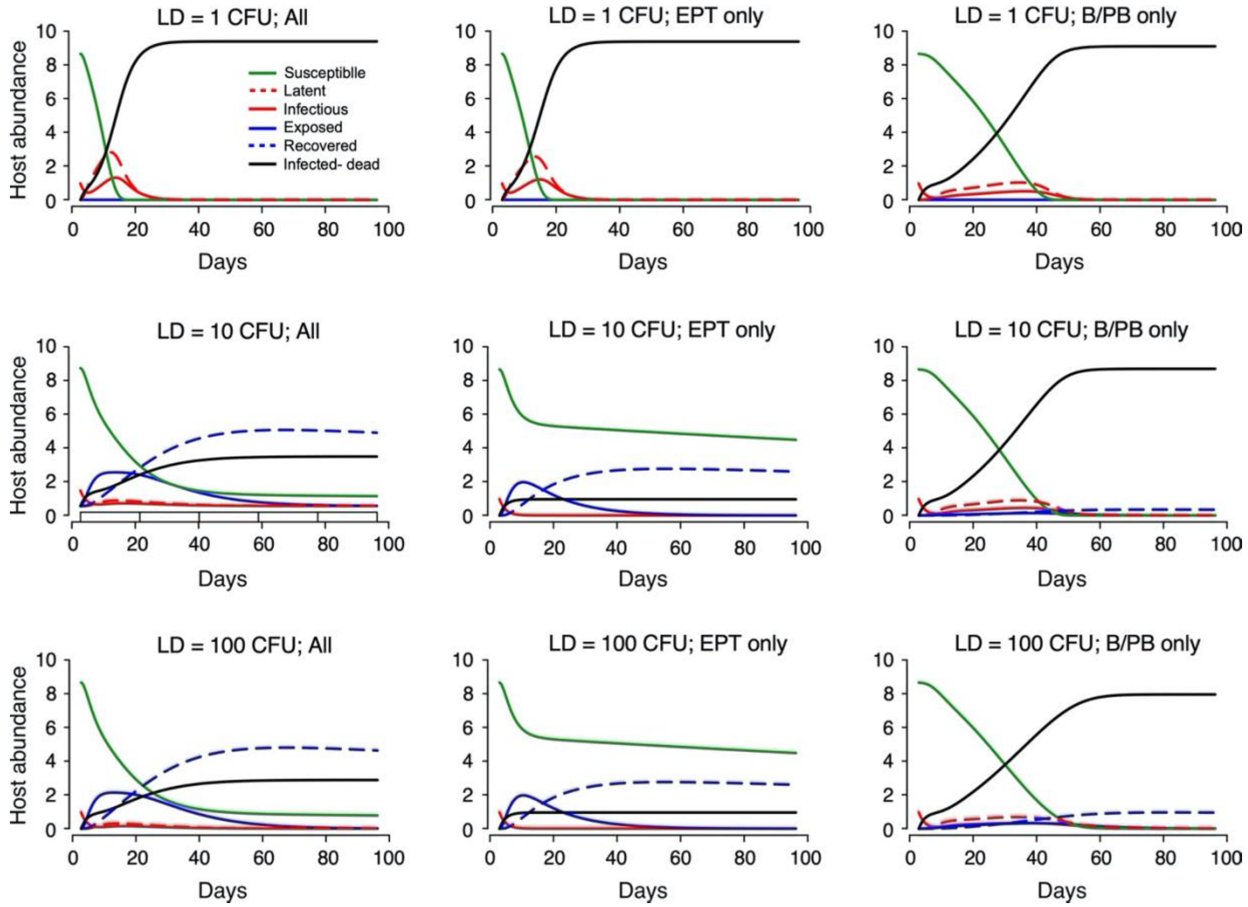

	Host outcome (%)		
Scenario (mouse blood)	Dead	Recovered	Susceptible
1 CFU; All	98	0	0
1 CFU; EPT only	98	0	0
1 CFU; B/PB only	95	0	0
10 CFU; All	32	48	6
10 CFU; EPT only	10	27	47
10 CFU; B/PB only	90	4	0
100 CFU; All	30	48	8
100 CFU; EPT only	10	27	47
100 CFU; B/PB only	83	10	0

Fig. S2B. Fleas infected using rat blood

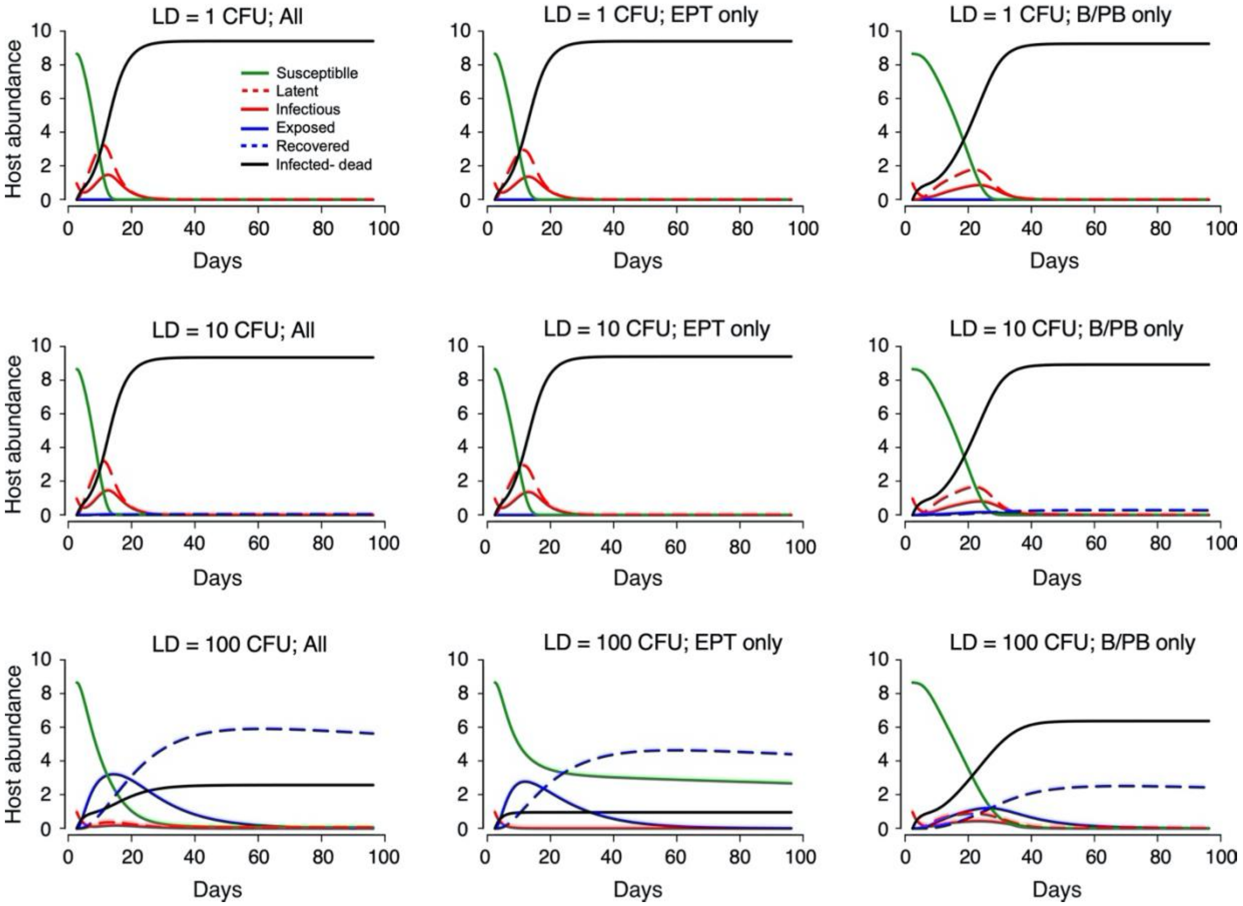

	Host outcome (%)		
Scenario (rat blood)	Dead	Recovered	Susceptible
1 CFU; All	98	0	0
1 CFU; EPT only	98	0	0
1 CFU; B/PB only	96	0	0
10 CFU; All	97	1	0
10 CFU; EPT only	98	0	0
10 CFU; B/PB only	93	3	0
100 CFU; All	27	59	1
100 CFU; EPT only	10	46	28
100 CFU; B/PB only	66	25	0

S2 Fig. Model output of the dynamics of plague in host populations with different levels of susceptibility [lethal dose (LD) of 1, 10, or 100 *Y. pestis* CFU], using the modified parameters for probability of transmission (*p*) and probability of transmission at or above a lethal dose (*t*) to account for cumulative transmission by simultaneous flea bites (Table 2). See text for details. Separate outcomes produced by fleas infected using mouse blood (**A**) or rat blood (**B**) in which both early-phase transmission and biofilm-dependent transmission by partially and completely blocked fleas are operative (All); or in which only early-phase transmission (EPT only) or only biofilm-dependent transmission (B/PB only) are operative are indicated. All simulations were initiated with 9 susceptible hosts, 1 infected (highly bacteremic) host, and 50 uninfected fleas. The percentage of infected-dead, infected-recovered, and uninfected, still susceptible hosts at the end of the 100-day simulation period and the R_0 values are shown in the Table.
