# Supplementary material for: A Role for Early-Phase Transmission in the Enzootic Maintenance of Plague
Source: PLoS Pathog. 2022 Dec 15;18(12):e1010996. doi: 10.1371/journal.ppat.1010996 (PMC9754260; doi:10.1371/journal.ppat.1010996)
Supplement: S3 Fig — (PDF) [file ppat.1010996.s004.pdf]

Fig. S3A. Fleas infected using mouse blood

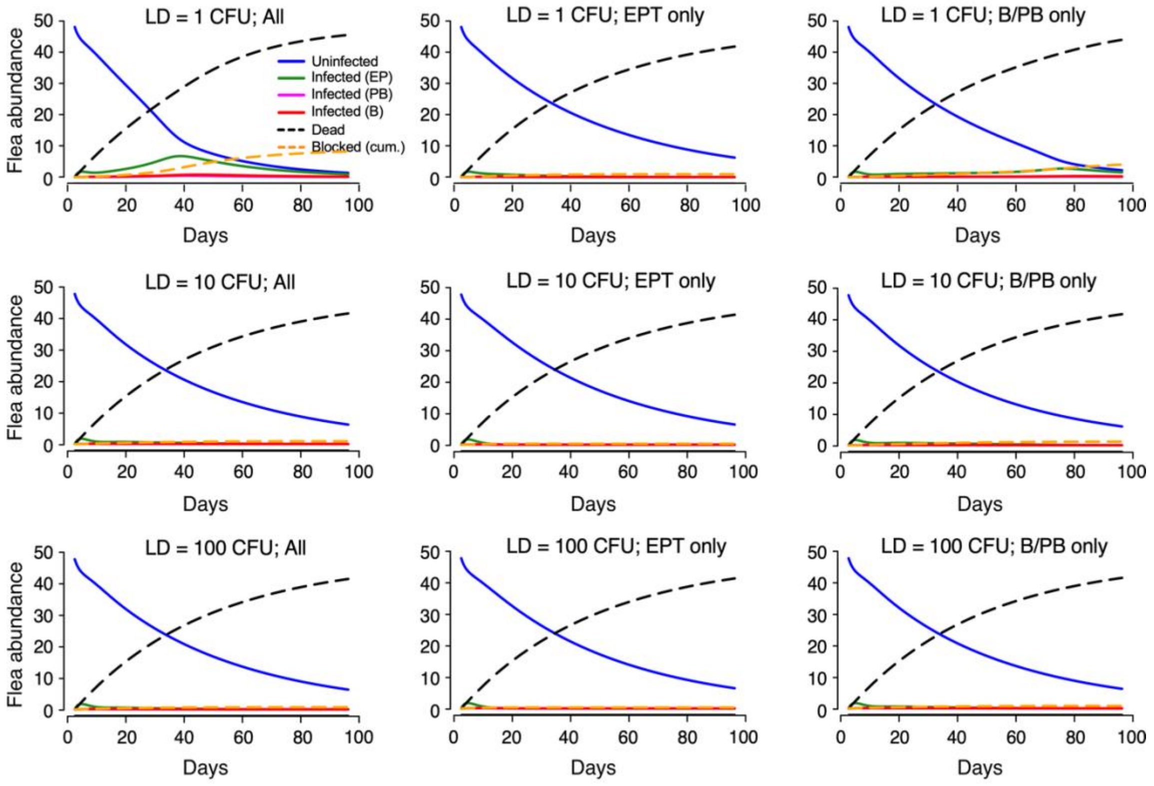

Fig. S3B. Fleas infected using rat blood

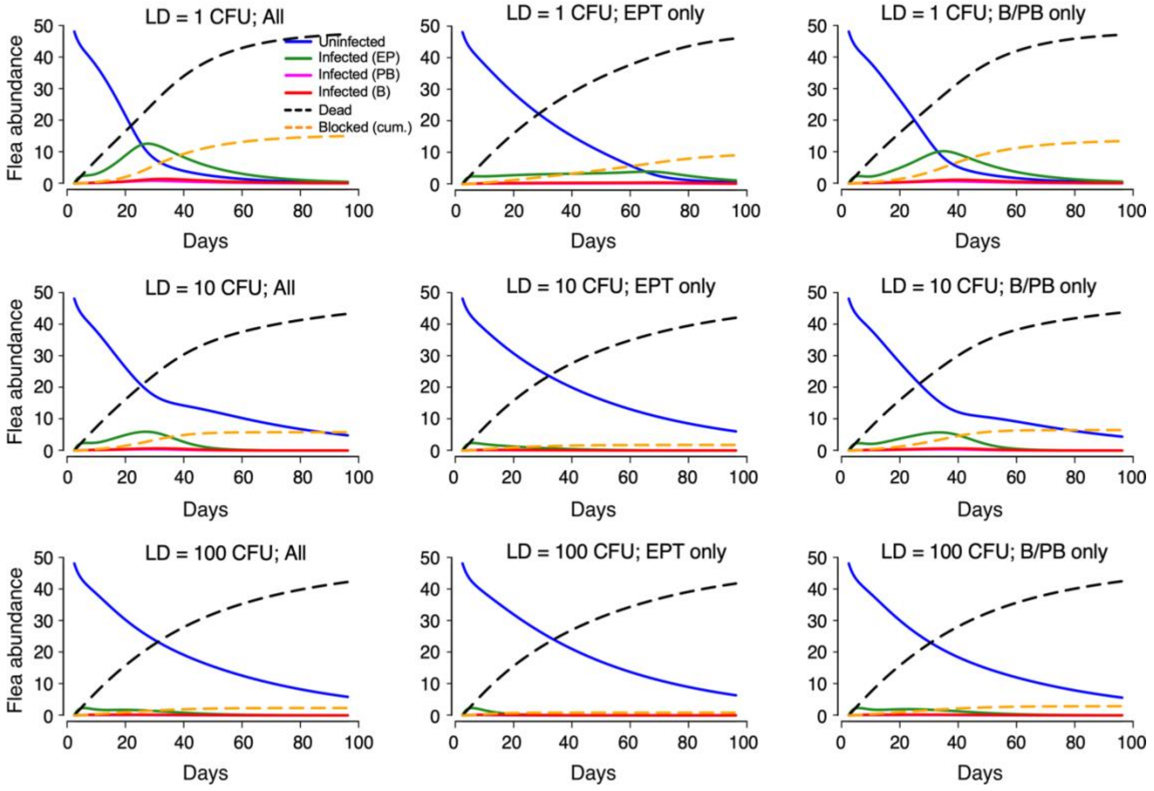

S3 Fig. Model output of the dynamics of flea infection, blockage, and mortality in scenarios with different levels of host susceptibility [lethal dose (LD) of 1, 10, or 100 *Y. pestis* CFU], using the unmodified parameters in Table 2. Separate outcomes produced by fleas infected using mouse blood (**A**) or rat blood (**B**) in which both early-phase transmission and biofilm-dependent transmission by partially and completely blocked fleas are operative (All); or in which only early-phase transmission (EPT only) or only biofilm-dependent transmission (B/PB only) are operative are indicated. All simulations were initiated with 9 susceptible hosts, 1 infected (highly bacteremic) host, and 50 uninfected fleas.
