# Supplementary material for: A Role for Early-Phase Transmission in the Enzootic Maintenance of Plague
Source: PLoS Pathog. 2022 Dec 15;18(12):e1010996. doi: 10.1371/journal.ppat.1010996 (PMC9754260; doi:10.1371/journal.ppat.1010996)
Supplement: S4 Fig — (PDF) [file ppat.1010996.s005.pdf]

Fig. S4A. Fleas infected using mouse blood

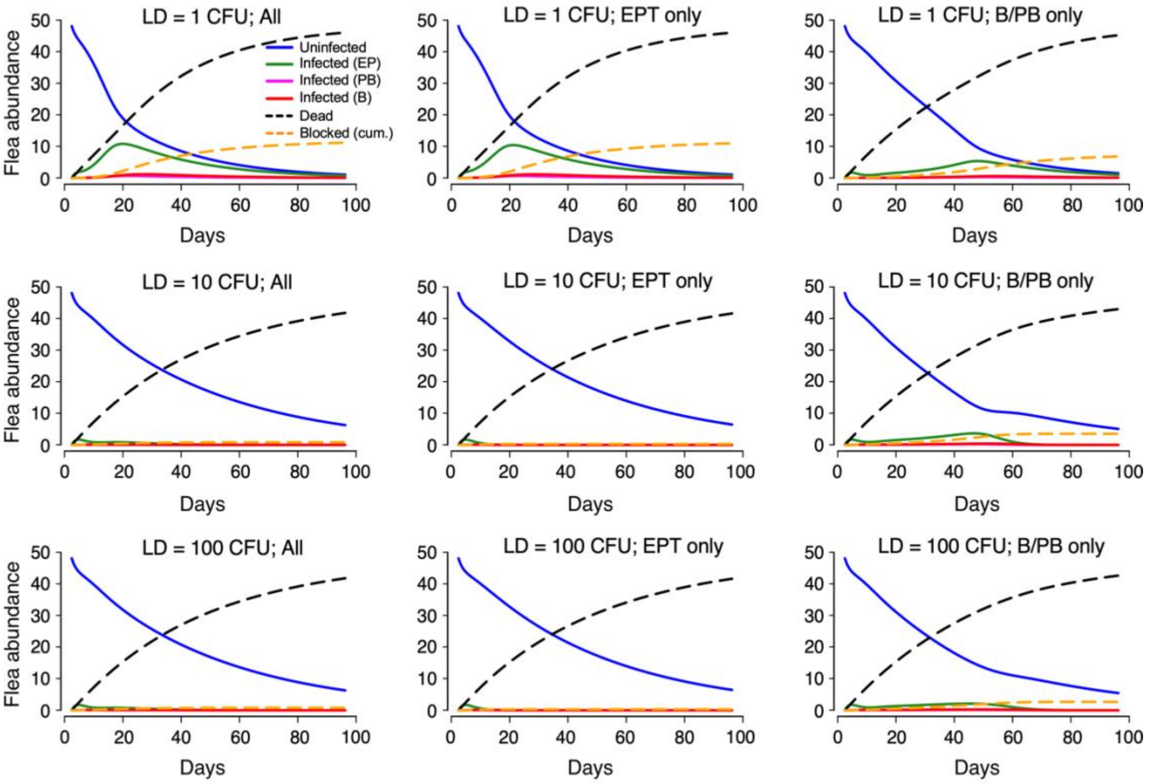

Fig. S4B. Fleas infected using rat blood

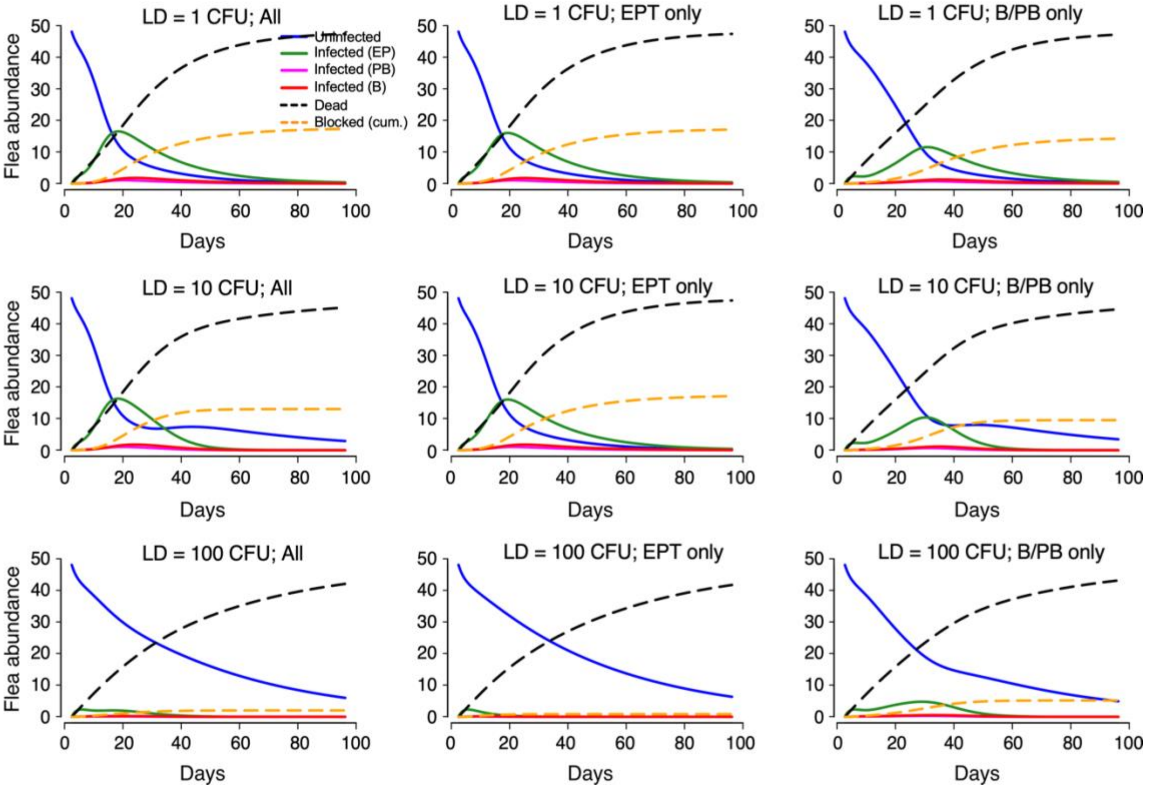

Fig. S4. Model output of the dynamics of flea infection, blockage, and mortality in scenarios with different levels of host susceptibility [lethal dose (LD) of 1, 10, or 100 *Y. pestis* CFU], using the modified parameters for probability of transmission (*p*) and probability of transmission at or above a lethal dose (*t*) to account for cumulative transmission by simultaneous flea bites (Table 2). See text for details. Separate outcomes produced by fleas infected using mouse blood (**A**) or rat blood (**B**) in which both early-phase transmission and biofilm-dependent transmission by partially and completely blocked fleas are operative (All); or in which only early-phase transmission (EPT only) or only biofilm-dependent transmission (B/PB only) are operative are indicated. All simulations were initiated with 9 susceptible hosts, 1 infected (highly bacteremic) host, and 50 uninfected fleas.
